# Species Limits and Hybridization in Andean Leaf‐Eared Mice (*Phyllotis*)

**DOI:** 10.1002/ece3.71783

**Published:** 2025-07-13

**Authors:** Marcial Quiroga‐Carmona, Schuyler Liphardt, Naim M. Bautista, Pablo Jayat, Pablo Teta, Jason L. Malaney, Tabitha McFarland, Joseph A. Cook, L. Moritz Blumer, Nathanael D. Herrera, Zachary A. Cheviron, Jeffrey M. Good, Guillermo D'Elía, Jay F. Storz

**Affiliations:** ^1^ School of Biological Sciences University of Nebraska Lincoln Nebraska USA; ^2^ Instituto de Ciencias Ambientales y Evolutivas, Facultad de Ciencias Universidad Austral de Chile Valdivia Chile; ^3^ Colección de Mamíferos, Facultad de Ciencias Universidad Austral de Chile Valdivia Chile; ^4^ Division of Biological Sciences University of Montana Missoula Montana USA; ^5^ Unidad Ejecutora Lillo (CONICET‐Fundación Miguel Lillo) San Miguel de Tucumán Argentina; ^6^ Departamento de Ciencias Básicas y Tecnológicas Universidad Nacional de Chilecito (UNdeC) La Rioja Argentina; ^7^ División Mastozoología, Museo Argentino de Ciencias Naturales “Bernardino Rivadavia” Ciudad Autónoma de Buenos Aires Buenos Aires Argentina; ^8^ New Mexico Museum of Natural History and Science Albuquerque New Mexico USA; ^9^ Museum of Southwestern Biology University of New Mexico Albuquerque New Mexico USA; ^10^ Department of Biology University of New Mexico Albuquerque New Mexico USA; ^11^ Department of Genetics University of Cambridge Cambridge UK

**Keywords:** Andes, introgression, Phyllotini, population genomics, Sigmodontinae, species delimitation

## Abstract

Leaf‐eared mice (genus *Phyllotis*) are among the most widespread and abundant small mammals in the Andean Altiplano, but species boundaries and distributional limits are often poorly delineated due to sparse survey data from remote mountains and high‐elevation deserts. Here, we report a combined analysis of mitochondrial DNA variation and whole‐genome sequence (WGS) variation in *Phyllotis* mice to delimit species boundaries, to assess the timescale of diversification of the group, and to examine evidence for interspecific hybridization. Estimates of divergence based on *cytb* data suggest that most diversification of *Phyllotis* occurred during the past 3 million years. Consistent with the Pleistocene Aridification hypothesis, our results suggest that diversification of *Phyllotis* largely coincided with climatically induced environmental changes in the mid‐ to late‐Pleistocene. Contrary to the Montane Uplift hypothesis, most diversification in the group occurred well after the major phase of uplift of the Central Andean Plateau. Species delimitation analyses revealed surprising patterns of cryptic diversity within several nominal forms, suggesting the presence of much undescribed alpha diversity in the genus. Results of genomic analyses revealed evidence of hybridization between the sister species *P. limatus* and *P. vaccarum*, suggesting that the contemporary zone of range overlap between the two species represents a hybrid zone.

## Graphical abstract

Diversification of Phyllotis coincided with mid‐ to late‐Pleistocene climate change. Species delimitation revealed cryptic diversity within several nominal forms. Genomic analyses revealed evidence for introgressive hybridization.

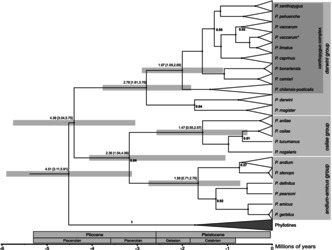

## 1 Introduction

Leaf‐eared mice in the genus *Phyllotis*, Waterhouse 1873, are emblematic mammals of the Andean Altiplano and have an exceptionally broad latitudinal distribution in South America, from Ecuador to the northern coast of the Strait of Magellan (Steppan and Ramírez 2015). The genus has an even more impressive elevational distribution: whereas *P. darwini* is found at sea level along the desert coastline of northern Chile, and species like *P. anitae* , *P. nogalaris* , and *P. osilae* are found in humid, lowland Yungas forests on the eastern sub‐Andean slopes (Jayat et al. 2016) an other taxa such as *P. vaccarum* have been documented at extreme elevations (> 6000 m above sea level) on the upper reaches and summits of some of the highest peaks in the Andean Cordillera (Storz et al. 2020, 2023, 2024; Steppan et al. 2022). Although *Phyllotis* mice are among the most abundant small mammals in the Andean Altiplano and adjacent lowlands, the taxonomic status and range limits of many forms are not well resolved due to sparse survey data from remote mountains and high‐elevation deserts (Andean Puna). The resultant gaps in sampling coverage have hindered a robust assessment of species richness and geographic distributions of *Phyllotis* mice and other sigmodontine rodents that are native to Andean highlands (Quiroga‐Carmona et al. 2023; Storz et al. 2024).

*Phyllotis* has been subject to several systematic assessments that have helped resolve species limits and phylogenetic relationships (Anderson and Yates 2000; Hershkovitz 1962; Jayat et al. 2007, 2016, 2021; Ojeda et al. 2021; Pearson 1958; Pearson and Patton 1976; Rengifo and Pacheco 2015, 2017; Salazar‐Bravo et al. 2013; Steppan et al. 2007; Teta et al. 2018, 2022). Most of these studies are focused on relatively narrow geographic regions and are based on morphological and/or mitochondrial variation (but see Storz et al. 2024). Notwithstanding, 23 species of *Phyllotis* are currently recognized (Mammal Diversity Database 2025), comprising three main clades, commonly referred to as the *andium‐amicus*, *osilae*, and *darwini* species groups (Steppan 1993, 1995; Steppan et al. 2007; Steppan and Ramírez 2015; Rengifo and Pacheco 2017; Teta et al. 2022). The *darwini* species group is the most speciose and includes several species that are distributed in the Atacama Desert and the Andean dry Puna: *P. caprinus* , “*P. chilensis‐posticalis*” (*sensu* Pearson 1958; referred to as “ *P. posticalis* ‐*rupestris*” by Ojeda et al. 2021), *P. darwini* , *P. limatus* , *P. magister* , *P. osgoodi* , and *P. vaccarum* (Jayat et al. 2021; Ojeda et al. 2021; Steppan and Ramírez 2015; Storz et al. 2024; Teta et al. 2022). With the exception of *P. darwini* , *P. magister* , and *P. osgoodi* , the above‐mentioned species belong to the *P. xanthopygus* complex, a set of closely related species whose common ancestry is supported by karyotypic (Walker et al. 1984) and molecular phylogenetic evidence (Ojeda et al. 2021; Steppan 1995, 1998; Steppan et al. 2007). However, there remains considerable uncertainty regarding species‐level distinctions among several nominal forms within the complex.

**FIGURE 1:**
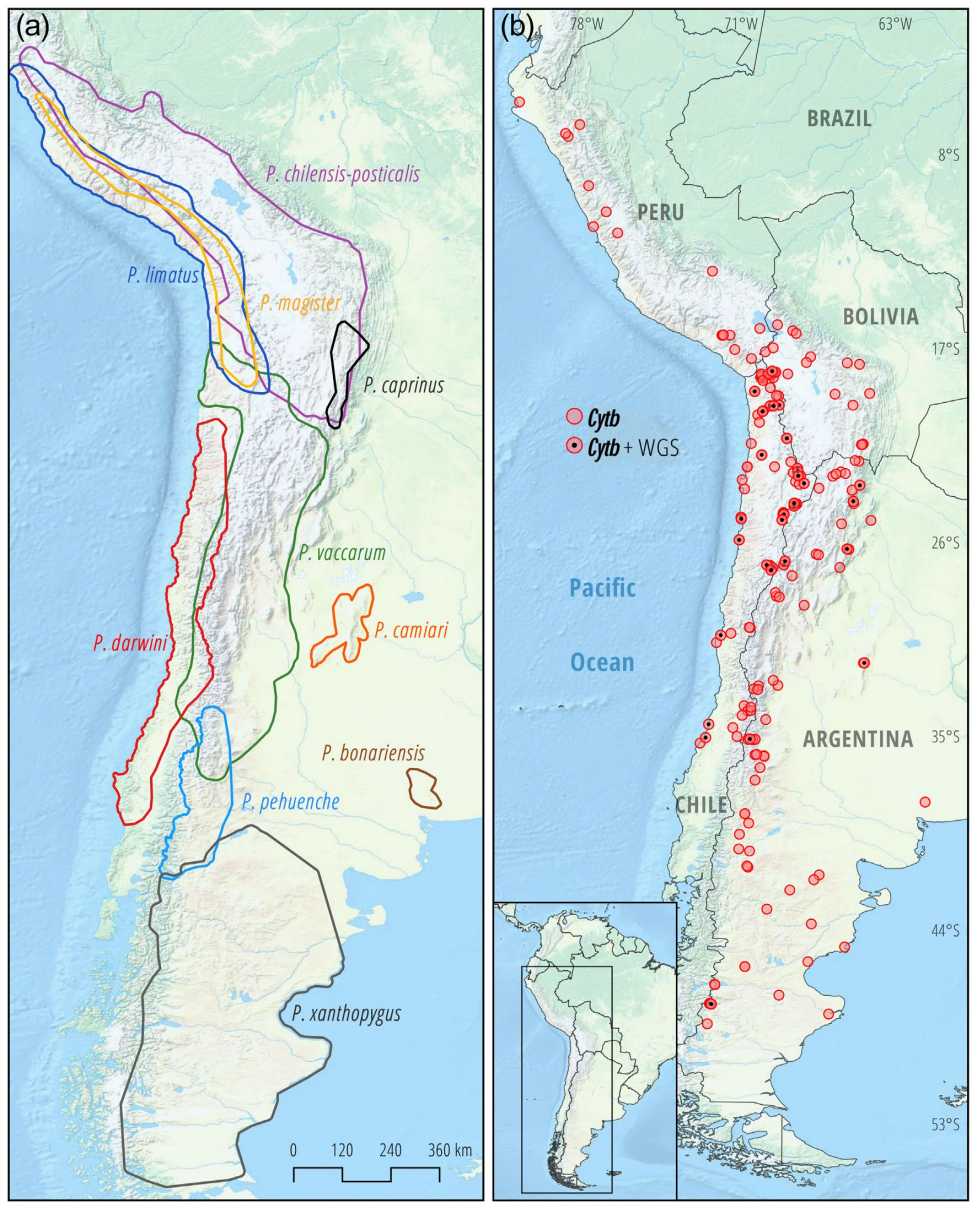
Distribution limits of *Phyllotis* species and geographic sampling coverage in the Central Andes and adjoining lowlands. (a) Inferred ranges of *Phyllotis* mice in the *P. darwini* species group, based on patterns of morphological and DNA marker variation (Jayat et al. 2021; Ojeda et al. 2021; Steppan and Ramírez 2015; Storz et al. 2024). (b) Distribution of 169 sampling localities, representing sites of origin for 448 *Phyllotis* specimens used in the survey of *cytb* (Table S1), and 137 specimens used to characterize WGS variation (Table S2). Collection localities are labeled according to whether they were a source of cytb samples only or were also characterized with WGS sampling.

In northern Chile and bordering regions of Argentina, Bolivia, and Peru, the ranges of several species in the *darwini* species group overlap (Figure 1a), but in most cases, the distribution limits are not clearly defined. For instance, we mostly do not know the extent to which species ranges overlap across Andean elevational gradients, which is important for understanding the relative roles of competitive exclusion and physiological tolerances in shaping elevational patterns of species turnover and for detecting distributional shifts in response to climate change (Novillo et al. 2024; Storz and Scott 2024).

Genomic delimitation of species boundaries between the sister species *Phyllotis limatus* and *P. vaccarum* in northern Chile led to a dramatically revised understanding of the latitudinal and elevational range limits of the former species (Storz et al. 2024). Previously inferred range limits of *P. limatus* were found to be in error because specimens from the highest elevations and most southern latitudes had been misidentified as *P. limatus* on the basis of mitochondrial (mt) DNA and were later referred to *P. vaccarum* on the basis of whole‐genome sequence data (Storz et al. 2024).

Here we report a combined analysis of mtDNA and whole‐genome sequence (WGS) variation in mice of the genus *Phyllotis* to delimit species boundaries, to assess the timescale of diversification of the group, and to examine evidence for interspecific hybridization between *P. limatus* and *P. vaccarum*. The analysis is principally focused on a large set of vouchered specimens that we collected over the course of five high‐elevation survey expeditions in the Puna de Atacama, Central Andes (2020–2023), in conjunction with additional collecting trips in the surrounding Altiplano and adjoining lowlands in Argentina, Bolivia, and Chile. The genomic analysis is primarily focused on members of the *P. darwini* species group that have overlapping or potentially overlapping ranges.

## 2 Materials and Methods

### 2.1 Specimen Collection

We collected representatives of multiple species of *Phyllotis* during the course of small‐mammal surveys in the Altiplano and adjoining lowlands on both sides of the Andean Cordillera in Chile, Bolivia, and Argentina. We captured all mice using Sherman live traps, in combination with Museum Special snap traps in some localities. We sacrificed animals in the field, prepared them as museum specimens, and preserved liver tissue in ethanol as a source of genomic DNA. Specimens are housed at Colección de Mamíferos de la Universidad Austral de Chile, Valdivia, Chile (UACH), Colección Boliviana de Fauna, La Paz, Bolivia (CBF), Centro Regional de Investigaciones Científicas y Transferencia Tecnológica de La Rioja, La Rioja, Argentina (CRILAR), Centro Nacional Patagónico, Chubut, Argentina (CNP), Fundación‐Instituto Miguel Lillo, Tucumán, Argentina (CML), Museo Argentino de Ciencias Naturales “Bernardino Rivadavia,” Ciudad Autónoma de Buenos Aires, Argentina (MACN‐Ma), and Museum of Southwestern Biology, New Mexico, USA (MSB). We identified all specimens to the genus level based on external characters (tail as long or longer than the head‐body length, medium to large ears with sparse inner hair and no ear patches, hindfeet with bare soles, six footpads and a reduced fifth toe; Jayat et al. 2021; Steppan and Ramírez 2015; Teta et al. 2022). As described below, we later confirmed field identifications with DNA sequence data.

In Chile, all animals were collected in accordance with permissions to JFS, MQC, and GD from the following Chilean government agencies: Servicio Agrícola y Ganadero (6633/2020, 2373/2021, 5799/2021, 3204/2022, 3565/2022, 911/2023, and 7736/2023), Corporación Nacional Forestal (171219, 1501221, and 31362839), and Dirección Nacional de Fronteras y Límites del Estado (DIFROL, Autorización de Expedición Científica #68 and 02/22). In Bolivia, all animals were collected in accordance with permissions to JFS (Resolución Administrativa 026/09) and JAC (DVS‐CRT‐02/91) from the Ministerio de Medio Ambiente y Agua, Estado Plurinacional de Bolivia. In Argentina, all animals were collected in accordance with the following permissions to JPJ from the Secretaría de Ambiente, Ministerio de Producción y Ambiente de La Rioja (Expte. N° P4‐00402‐21 Disp. S.A. N° 001/22 and Expte. N° P4‐00158‐22 Disp. S.A. N° 007/22), the Ministerio de Ambiente, Secretaría de Biodiversidad y Desarrollo Sustentable de Jujuy (Expte. N° 1102–122‐2020/SByDS), and the Ministerio de Desarrollo Productivo, Dirección de Flora, Fauna Silvestre y Suelos de Tucumán (Expte. N° 677‐330‐2021). All live‐trapped animals were handled in accordance with protocols approved by the Institutional Animal Care and Use Committee (IACUC) of the University of Nebraska (project ID's: 1919, 2100), IACUC of the University of New Mexico (project ID's: 16787 and 20405), and the bioethics committee of the Universidad Austral de Chile (certificate 456/2022).

### 2.2 Sequence Data

To maximize geographic coverage in our survey of mtDNA variation, we generated sequence data for a subset of our own voucher specimens (*n* = 269) and supplemented this dataset with publicly available *Phyllotis* sequences from GenBank (*n* = 179). This sequence dataset (Table S1), based on a total of 448 specimens, includes 20 of the 23 nominal species that are currently recognized within the genus *Phyllotis*. The taxonomic status of *P. osgoodi* is unclear, and recent taxonomic revisions have cast doubt on its distinction (Steppan 1998; Steppan et al. 2007; Steppan and Ramírez 2015). We lack representatives of this nominal form. To complement the analysis of mtDNA variation, we used a subset of our newly collected voucher specimens (*n* = 137) for the analysis of WGS variation (Figure 1b, Table S2).

### 2.3 Mitochondrial DNA Variation

For the analysis of mtDNA variation, we extracted DNA from liver samples and PCR‐amplified the first 801 base pairs of the *cytochrome b* (*cytb*) gene using the primers MVZ 05 and MVZ 16 (Smith and Patton 1993), following protocols of Cadenillas and D'Elía (2021). Of the 269 *cytb* sequences that we generated from our own set of voucher specimens, 89 were published previously (Storz et al. 2020, 2024; GenBank accession numbers: OR784643‐OR784661, OR799565‐OR799614, and OR810731‐OR810743). We deposited all newly generated sequences in GenBank (accession numbers: PQ295377–PQ295555). The newly generated sequences derive from voucher specimens housed in the Argentine, Bolivian, Chilean, and US collections mentioned above (Section 2.1).

### 2.4 Phylogeny Estimation

As outgroups for the phylogenetic analysis, we used *cytb* sequences from five other phyllotine rodents ( *Auliscomys boliviensis* , JQ434420; *A. pictus* , U03545; *A. sublimis* , U03545; *Calomys musculinus* , HM167822; and *Loxondontomys micropus*, GU553838). The final set of 453 sequences was aligned with MAFFT v7 (Katoh et al. 2017) using the E‐INS‐i strategy to establish character primary homology. The aligned matrix was visually inspected with AliView v1.26 (Larsson 2014) to check for the presence of internal stop codons and shifts in the reading frame. Pairwise genetic distances and their standard errors (p‐dist./SE) were calculated using MEGA X 10.1.8 (Kumar et al. 2018). After first recording the geographic distribution and phylogenetic placement of all *cytb* sequences (Table S1), we then identified and excluded redundant haplotypes using the functions *FindHaplo* and *haplotype* in the *sidier* (Pajares 2013) and *haplotypes* (Aktas 2023) R packages, respectively. These packages and the following ones were implemented in R v 4.0.0 (R Core Team 2020). The final matrix of nonredundant sequences included a total of 287 haplotypes.

We selected the nucleotide substitution model (HKY + I + G) because it provided the best fit to the nonredundant *cytb* data matrix according to the Bayesian Information Criterion (BIC; computed using ModelFinder; Kalyaanamoorthy et al. 2017). Genealogical relationships among haplotypes of *Phyllotis* species were estimated via Maximum Likelihood (ML) and Bayesian Inference (BI). The ML analysis was performed using IQ‐TREE (Trifinopoulos et al. 2016), with perturbation strength set to 0.5 and the number of unsuccessful iterations set to 100. Nodal support was assessed through 1000 ultrafast bootstrap replicates (UF; Minh et al. 2013). BI was implemented with BEAST 2 v2.6.7 (Bouckaert et al. 2014), which was also used to estimate divergence dates among *Phyllotis* species. A gamma site model was selected with the substitution model set to HKY. The gamma shape parameter (exponential prior, mean 1.0) and proportion of invariant sites (uniform distribution, 0.001–0.999, lower and upper bounds) were estimated. To prevent the sampling of excessively small values for the HKY exchangeability rates, the prior sampling distribution was set to gamma with a shape parameter (alpha) of 2.0 and a scale parameter (beta) of 0.5. The clock model was set to Relaxed Log Normal with an estimated clock rate. The calibrated Yule model was selected to parameterize fossil calibrations. For the mean branch rate (ucldMean), an exponential sampling distribution was applied with a mean of 10.0 and no offset. Given that variation in substitution rates among branches is low and evidence suggests that molecular evolution is largely clock‐like across Phyllotini (Parada et al. 2013), standard deviation in rates across branches (ucldStdev) was converted to an exponential prior distribution with a mean of 0.3337 and no offset. Since the fossil record for *Phyllotis* is not sufficient to establish primary calibration points (Pardiñas et al. 2002), we used secondary calibration points from a phylogenetic analysis of the subfamily Sigmodontinae (Parada et al. 2015). We used normal distributions and 95% credibility intervals for estimated crown ages of the genus *Phyllotis* (3.35–6.66 Mya) and the *darwini* species group (4.51–1.77 Mya). We performed two runs of 600 × 10^6^ MCMC generations with trees sampled every 4 × 10^3^ steps, yielding 15,001 samples for parameter estimates. Effective sample sizes greater than 200 for all parameters (i.e., stable values of convergence) were verified using Tracer v1.7.1 (Rambaut et al. 2018). Runs were combined with LogCombiner v2.6.7 (Bouckaert et al. 2014), using a 10% burn‐in that was determined by examining individual traces. The first 10% of estimated trees were discarded and the remainder were used to construct a maximum clade credibility tree with posteriori probability values (PP) and age estimates employing TreeAnnotator v2.6.2 (Rambaut and Drummond 2019).

### 2.5 Assessment of Species Limits Within the *P. xanthopygus* Species Complex

To delimit species within the *P. darwini* group, we employed the Bayesian time calibrated‐ultrametric tree estimated with BEAST 2 and two single‐locus coalescent methods: the General Mixed Yule Coalescent model (GMYC; Pons et al. 2006; Fujisawa and Barraclough 2013) and the Poisson Tree Processes (PTP; Zhang et al. 2013). Both methods are based on the fit of different mixed models (the General Mixed Yule Coalescent model in the case of the GMYC, and the Poisson Tree Processes in the case of the PTP) to processes of interspecific diversification and/or genealogical branching within species (Fujisawa and Barraclough 2013; Zhang et al. 2013). These methods were implemented via their online web servers: https://species.h‐its.org/gmyc/ and http://species.h‐its.org/ptp/, respectively. The Bayesian implementations of these methods (b‐GMYC: Reid and Carstens 2012; b‐PTP: Zhang et al. 2013) were also employed to account for uncertainty in gene tree estimation. The b‐GMYC analysis was implemented in R via the *b‐GMYC* R package (Reid and Carstens 2012), which offers estimates of the posterior marginal probabilities for candidate species, setting a post‐burn‐in sample of 1000 trees sampled from the posterior distribution of trees. For all parameters, priors were set as default (i.e., t1 and t2 were set at 2 and 100, respectively), and the analysis was completed with 50 × 10^3^ generations, burning 10% of these and with a thinning interval of 1000 samples. The b‐PTP analysis was implemented in the associated online web server (http://species.h‐its.org/b‐ptp/) with default values (i.e., 100 × 10^3^ MCMC, thinning of 100 and burning of 0.1). Branch lengths are proportional to coalescence times in the GMYC model, whereas they are proportional to the number of nucleotide substitutions in the PTP model (Dellicour and Flot 2018).

### 2.6 Whole‐Genome Sequence Data

We generated low‐coverage whole‐genome sequence (WGS) data for a subset of 137 *Phyllotis* specimens that were included in the *cytb* data matrix, which we analyzed in conjunction with a chromosome‐level reference genome for *Phyllotis vaccarum* (Storz et al. 2023). Depth of coverage ranged from 1.04× to 24.06× (median = 2.58×). According to field identifications and *cytb* haplotypes, this set of specimens represented a total of 11 species ( *P. anitae* , *P. camiari*, *P. caprinus* , *P. chilensis* , *P. darwini* , *P. limatus* , *P. magister* , *P. nogalaris* , *P. pehuenche*, *P. vaccarum*, and *P. xanthopygus* ), several of which have potentially overlapping ranges (Figure 1a). All species other than *P. anitae* and *P. nogalaris* are members of the *darwini* species group. Of the 137 vouchered specimens included in the genomic analysis, data for 61 specimens representing *P. chilensis* , *P. limatus* , *P. magister* , and *P. vaccarum* were published previously (Storz et al. 2024). The WGS dataset is available in NCBI SRA, under BioProject code PRJNA950396.

#### 2.6.1 Genomic Library Preparation and Whole‐Genome Sequencing

All library preparations for whole genome resequencing experiments were conducted in the University of Montana Genomics Core facility. We extracted genomic DNA from ethanol‐preserved liver tissue using the DNeasy Blood and Tissue kit (Qiagen). We used a Covaris E220 sonicator to shear DNA, and we then prepared genomic libraries using the KAPA HyperPlus kit (Roche). Individual libraries were indexed using KAPA UDI's, and pooled libraries were sent to Novogene for Illumina paired‐end 150 bp sequencing on a Novaseq X.

#### 2.6.2 Read Quality Processing and Mapping to the Reference Genome

We used fastp 0.23.2 (Chen et al. 2018) to remove adapter sequences, and to trim and filter low‐quality reads from sequences generated from library preparations. We used a 5 bp sliding window to remove bases with a mean quality less than 20, and we discarded all reads < 25 bp. We merged all overlapping reads that passed filters and retained all reads that could not be merged or whose paired reads failed filtering. We separately mapped merged reads, unmerged but paired reads, and unpaired reads to the *P. vaccarum* reference genome with BWA 0.7.17 (Li and Durbin 2009) using the mem algorithm with the ‐M option. We sorted, merged, and indexed all resulting binary alignment maps with SAMtools 1.15.1 (Li et al. 2009) and used picard 2.27.4 to detect and remove PCR duplicates. We used GATK 3.8 (McKenna et al. 2010) to perform local realignment around targeted indels, and to generate the final BAM files used in subsequent analyses.

#### 2.6.3 Mitochondrial Genome Assembly

A *de novo* assembly of the mitochondrial genome of *Phyllotis vaccarum* (specimen UACH8291) as a seed sequence, we used NOVOplasty 4.3.3 (Dierckxsens et al. 2017) to generate *de novo* mitochondrial genome assemblies for all other *Phyllotis* specimens. We annotated assembled mitochondrial genomes with MitoZ to identify coding sequences, and we generated a multiple alignment of coding sequences with MAFFT 7.508 (Katoh and Standley 2013), using the ‐‐auto flag to determine the best algorithm given the data.

### 2.7 Analysis of Whole‐Genome Sequence Variation in *Phyllotis*

First, we randomly downsampled all higher coverage samples to the median coverage (2.58×) using SAMtools 1.17 to avoid artifacts associated with variation in coverage across samples that can impact inferences of population structure. We calculated genotype likelihoods for scaffolds 1–19 (covering > 90% of the *Phyllotis* genome) for all samples in ANGSD 0.939 (Korneliussen et al. 2014). We used ‐GL 2 to specify the GATK model for genotype likelihoods, retained only sites with a probability of being variable > 1e‐6 with ‐SNP_pval 1e‐6. We filtered out nonuniquely mapped reads with ‐remove_bads 1 and ‐uniqueOnly 1, respectively, and only retained reads and bases with a mapping quality higher than 20. We adjusted mapping quality for excessive mismatches with ‐C 50. We used PCAngsd v.0.99.0 (Meisner and Albrechtsen 2018) to calculate the covariance matrix from genotype likelihoods and used a minor allele frequency filter of 0.05. Finally, we calculated eigenvectors and plotted the first, second, and third principal components using the R package *ggplot2* (Wickham 2016).

Based on the results of our genus‐wide genomic PCA, we recalculated genotype likelihoods and performed additional genomic analyses on a subset of *Phyllotis vaccarum* and *P. limatus* specimens (*n* = 51 and 20, respectively). To test for admixture between *P. vaccarum* and *P. limatus* , we calculated ancestry proportions with NGSadmix (Skotte et al. 2013). This analysis included all *P. vaccarum* specimens, including those with *limatus*‐like *cytb* haplotypes. To alleviate computational costs associated with NGSadmix, we generated a reduced SNP set by sampling every hundredth SNP calculated by ANGSD. We ran NGSadmix with *K* = 1–10, with 10 iterations for each *K* value with a random starting seed and a minor allele frequency filter of 0.05. We evaluated the optimal *K* value using EvalAdmix 0.95, which calculates the pairwise covariance matrix of residuals of model fit. The results of EvalAdmix determined *K* = 2 as the optimal value of *K*. We combined individual runs for each *K* value with the R package PopHelper 2.3.1 to average estimates of ancestry across runs.

In comparisons between species, we calculated log‐transformed p‐distances using ngsDist v1.0.10 (Vieira et al. 2015). We calculated genotype likelihoods for the full WGS data set using ANGSD, as described previously, but with invariant sites included to make estimated distances comparable to those estimated from the mtDNA sequence data. We extracted five million base pairs from the beginning of scaffold one, excluding the first five million base pairs to avoid the telomeric repeat portion (i.e., positions 5 × 10^6^–10 × 10^6^) to make our analysis computationally feasible. Mean interspecific p‐distances were calculated using a custom python script.

### 2.8 Genomic Patterning of Admixture

To examine the genomic patterning of mixed *P. vaccarum*/ *P. limatus* ancestry, we conducted a windowed PCA of nucleotide variation. We used the script windowed_pcangsd.py (https://doi.org/10.5281/zenodo.8127993) to compute the first principal component in 90% overlapping 1 Mbp windows along chromosomes 1–19, using the subset of 51 *P. vaccarum* and 20 *P. limatus* samples and employing a minor allele frequency threshold of 0.01. For visualization, we excluded outlier windows (those with less than 0.3% informative sites and those featuring the largest 0.005% absolute PC1 values across the genome). For consistency, we polarized PC1 orientation by its sign for chromosome 1 since polarity is arbitrary in principal component analyses.

## 3 Results

The *cytb* sequence data derive from a total of 448 specimens of *Phyllotis* from 169 localities that span most of the distributional range of the genus (Figure 1b). For the analysis of WGS variation, we used a subset of 137 vouchered specimens representing 11 nominal species of *Phyllotis* that have overlapping or potentially overlapping ranges in Argentina, Bolivia, and Chile. *P. vaccarum* is one of the most broadly distributed species in this region, and different parts of its current or historic range potentially overlap with those of *P. caprinus* , *P. chilensis‐posticalis*, *P. darwini* , *P. limatus* , *P. magister* , and *P. pehuenche* (Figure 1a). We therefore concentrated much of our sampling efforts on these zones of range overlap to examine evidence of introgressive hybridization.

### 3.1 Phylogenetic Relationships and Divergence Times

**FIGURE 2:**
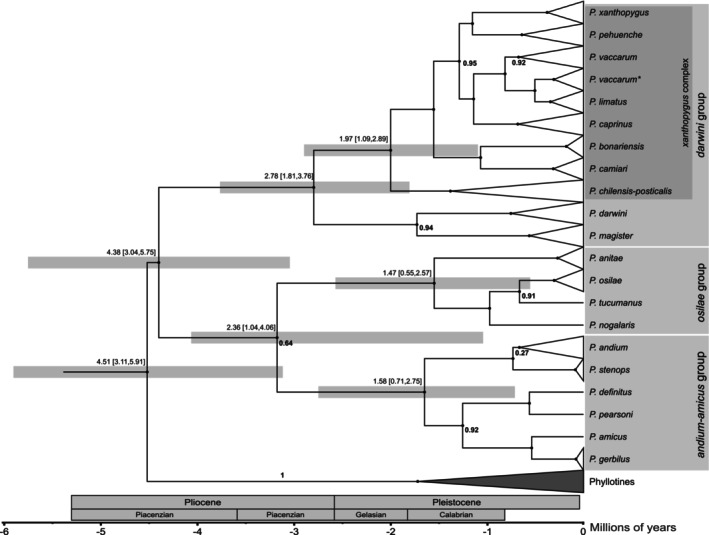
Calibrated maximum clade credibility tree showing Bayesian estimates of phylogenetic relationships and divergence times within the genus *Phyllotis*. Estimates of the 95% Highest Posterior Distributions interval for the divergence times are shown for main clades. Node support is shown only for those cases in which Bayesian posterior probability values were < 1. Specimens in the clade labeled “P. vaccarum*” carry *cytb* haplotypes that group with haplotypes of *P. limatus* , even though whole‐genome sequence data confirmed their identity as *P. vaccarum* (Storz et al. 2024).

Phylogeny estimates based on BI and ML both recovered three main clades within *Phyllotis* corresponding to the *andium‐amicus*, *osilae*, and *darwini* species groups (Figure 2, Figure S1). In the BI analysis, clades of the *andium‐amicus* and *osilae* species groups were recovered as sister groups (Bayesian Posterior Probability [PP] = 0.64) (Figure 2), whereas the ML analysis conversely placed the *osilae* species group as sister to a weakly supported clade (Bootstrap Percentage [BP] = 53) formed by the *andium‐amicus* and *darwini* species groups (Figure S1). Within the *darwini* species group, BI and ML analyses generally recovered the same set of relationships within the *P. xanthopygus* complex, with the exception that the BI phylogeny placed *P. pehuenche* and *P. xanthopygus* as sister (PP = 1; Figure 2), whereas the ML phylogeny placed *P. xanthopygus* as sister to the weakly supported clade containing *P. caprinus* , *P. limatus* , *P. vaccarum*, and *P. pehuenche* (BP = 70; Figure S1).

The median estimated crown age for the genus *Phyllotis* was 4.51 Mya with a 95% Highest Posterior Distribution (HPD) of 3.11–5.91 Mya, a range that spans nearly the entire Pliocene. Crown ages and associated HPD's for the clades corresponding to the species groups *andium‐amicus*, *osilae*, and *darwini* were 1.58 (0.71–2.75), 1.47 (0.55–2.57), and 2.78 Mya (1.81–3.76), respectively. Within each of these three groups, most species diverged during the last ~2 Mya and there appears to have been a pulse of speciation during the mid‐ to late‐Pleistocene.

**FIGURE 3:**
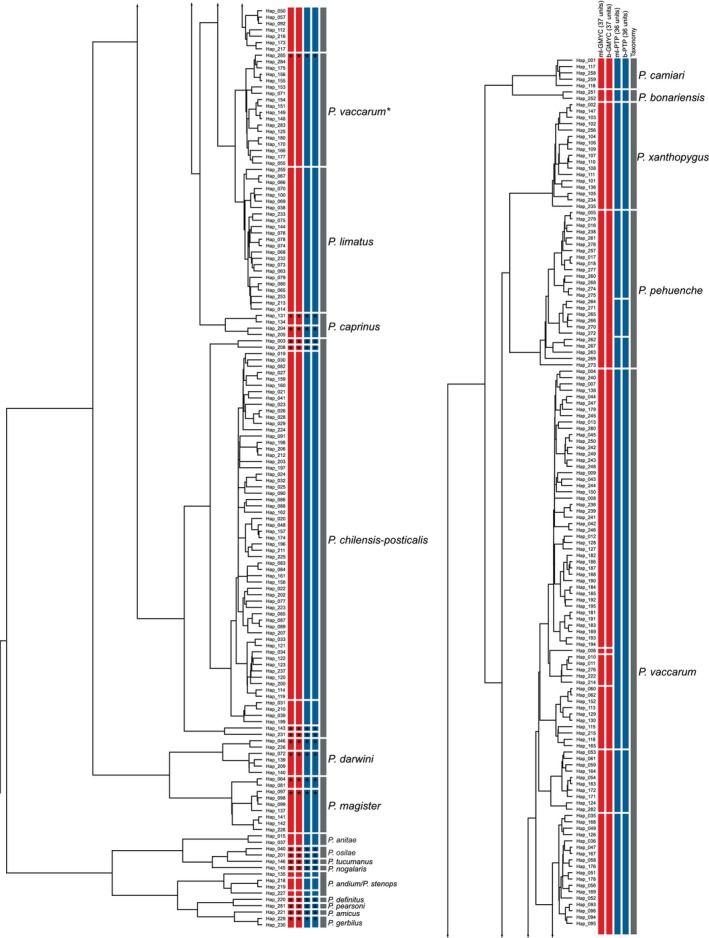
Maximum clade credibility depicting the delimitation schemes inferred from GMYC (red bars) and PTP (blue bars). Gaps in the vertical bars denote units delimited by each method, and asterisks denote splits with support values > 0.75. Continuous gray bars denote current taxonomic designations for nominal species. Terminal labels depict the haplotype classes of sequences that were retained to construct the nonredundant matrix of *cytb* haplotypes. Specimens in the clade labeled “P. vaccarum*” carry *cytb* haplotypes that group with haplotypes of *P. limatus* , even though whole‐genome sequence data confirmed their identity as *P. vaccarum* (Storz et al. 2024).

The species delimitation analyses were consistent in recognizing each of the 20 *Phyllotis* species represented in the full *cytb* dataset. Different delimitation approaches identified 36–37 distinct candidate species (Figure 3). Results of the delimitation analyses suggest that *P. caprinus* , *P. chilensis* ‐*posticalis*, *P. darwini* , *P. magister* , and *P*. *vaccarum* may each represent complexes of multiple species. The internal subdivisions identified within *P. caprinus* and *P. darwini* , and some of those identified within *P. chilensis‐posticalis*, have allopatric distributions (Figure S2). Results of the GMYC and PTP delimitation analyses differed in the number of units identified within *P*. *vaccarum* and *P*. *pehuenche*. The GMYC and b‐GMYC analyses identified six distinct units within *P*. *vaccarum* and recognized *P*. *pehuenche* as a single unit. By contrast, the PTP and b‐PTP approaches recognized three distinct units within both *P*. *vaccarum* and *P*. *pehuenche*.

Levels of *cytb* sequence differentiation between pairs of *Phyllotis* species are highly variable, with estimated *p*‐distances ranging from 2.73% (SE = 0.396) between *P. limatus* and *P*. *vaccarum*, to 17.28% (SE = 1.49) between *P*. *gerbilus* and *P. nogalaris* (Table 1). The mean *p*‐distance between nominal species within the genus *Phyllotis* is 7.55% (SE = 0.152). Within the *P. xanthopygus* species complex, the maximum *p*‐distance is 10.82% between *P*. *pehuenche* and *P. chilensis‐posticalis* (Table 1). We also estimated *p*‐distances between clades within species (candidate species). In these cases, pairwise *p*‐distances ranged from 1.81% (SE = 0.34) between subdivisions within *P. magister* to 8.99% (SE = 0.98) between the most divergent subdivisions within *P. chilensis‐posticalis* (Table S3).

**TABLE 1:** Mean *cytb p*‐distances between pair of species of *Phyllotis* (below diagonal).

	1	2	3	4	5	6	7	8	9	10	11	12	13	14	15	16	17	18	19	20
1. *P. amicus*	**—**	0.998	1.221	1.205	1.204	1.178	1.241	1.219	1.011	1.114	1.255	1.289	1.223	1.154	1.165	1.050	1.121	1.215	1.159	1.303
2. *P. andium*	11.498	**5.381**	1.193	1.193	1.195	1.082	1.075	1.061	1.111	1.125	1.049	1.260	1.138	0.920	1.234	1.112	0.559	1.286	1.120	1.134
3. *P. anitae*	14.414	12.032	**1.253**	1.335	1.237	1.355	1.166	1.347	1.398	1.387	1.259	1.083	1.078	1.156	1.482	1.322	1.224	1.071	1.366	1.343
4. *P. bonariensis*	15.855	13.615	14.052	**0.749**	0.877	0.864	1.044	1.238	1.180	0.978	0.964	1.276	1.252	1.132	0.879	1.022	1.140	1.315	0.886	0.880
5. *P. camiari*	14.657	13.048	12.119	8.514	**1.049**	0.886	1.000	1.244	1.256	1.036	0.986	1.214	1.225	1.160	1.001	1.019	1.306	1.290	0.988	0.938
6. *P. caprinus*	16.062	13.625	13.616	8.704	9.218	**4.061**	1.072	1.207	1.260	0.822	1.034	1.288	1.178	1.146	0.911	0.877	1.215	1.148	0.787	0.914
7. *P. darwini*	15.844	13.466	13.597	12.196	12.774	12.469	**3.305**	1.334	1.318	1.020	1.062	1.254	1.198	1.170	1.116	1.133	1.235	1.178	1.016	1.079
8. *P. definitus*	12.453	10.873	14.994	15.105	15.341	15.807	15.459	**0.001**	1.280	1.269	1.460	1.347	1.391	0.995	1.354	1.385	1.125	1.327	1.232	1.234
9. *P. gerbilus*	6.173	12.031	15.722	15.432	15.240	15.931	16.540	12.638	**0.274**	1.211	1.302	1.486	1.385	1.236	1.135	1.174	1.269	1.270	1.219	1.337
10. *P. limatus*	14.237	12.813	13.227	8.636	9.014	7.250	11.946	14.827	14.435	**0.512**	1.021	1.350	1.324	1.052	0.976	0.999	1.284	1.283	0.396	1.046
11. *P. magister*	14.915	12.630	13.351	10.609	10.575	11.008	10.852	14.961	15.877	9.711	**1.568**	1.139	1.082	1.176	1.030	1.002	1.125	1.135	0.976	0.956
12. *P. nogalaris*	16.105	14.206	11.259	14.232	14.157	16.030	15.114	15.698	17.284	14.566	14.328	**—**	1.035	1.146	1.373	1.352	1.302	1.067	1.296	1.320
13. *P. osilae*	14.723	12.551	10.068	14.082	13.900	14.708	15.349	15.721	16.283	14.043	13.301	10.205	**3.125**	1.158	1.331	1.252	1.185	0.910	1.309	1.197
14. *P. pearsoni*	12.406	9.862	13.181	14.286	14.361	15.353	14.387	7.103	12.948	13.878	13.234	15.664	14.210	**—**	1.214	1.118	0.952	1.202	0.986	1.158
15. *P. pehuenche*	15.874	14.086	14.730	9.277	10.602	9.128	13.325	16.252	16.280	8.894	11.256	15.689	15.516	15.499	**1.449**	0.950	1.270	1.279	0.961	1.009
16. *P. chilensis‐posticalis*	15.236	14.469	14.023	9.507	9.632	9.674	12.576	16.412	15.647	9.095	10.992	14.492	15.134	14.687	10.820	**1.578**	1.180	1.172	0.980	0.997
17. *P. stenops*	11.857	4.801	11.710	13.111	12.797	13.393	13.883	11.093	12.250	12.668	11.744	14.680	11.732	10.056	14.407	14.184	**0.252**	1.289	1.266	1.246
18. *P. tucumanus*	14.591	12.660	10.032	13.836	13.182	14.074	14.370	14.571	15.768	14.041	12.413	10.189	6.811	13.962	15.467	14.241	11.909	**—**	1.328	1.338
19. *P. vaccarum*	15.351	13.194	14.289	8.512	9.186	7.304	12.260	15.270	15.453	2.733	10.171	14.894	14.756	13.973	9.170	9.513	13.164	14.946	**2.224**	0.981
20. *P. xanthopygus*	15.205	12.941	14.237	8.010	9.383	8.304	11.199	15.837	15.032	8.668	10.461	14.008	13.467	14.902	9.540	10.279	12.910	13.890	8.737	**0.829**

*Note:* Mean values for intraspecific *p*‐distances are shown in bold on the diagonal. Standard errors (SE) for each estimate of pairwise distance is shown above the diagonal.

### 3.2 Genomic Assessment of Species Limits

**FIGURE 4:**
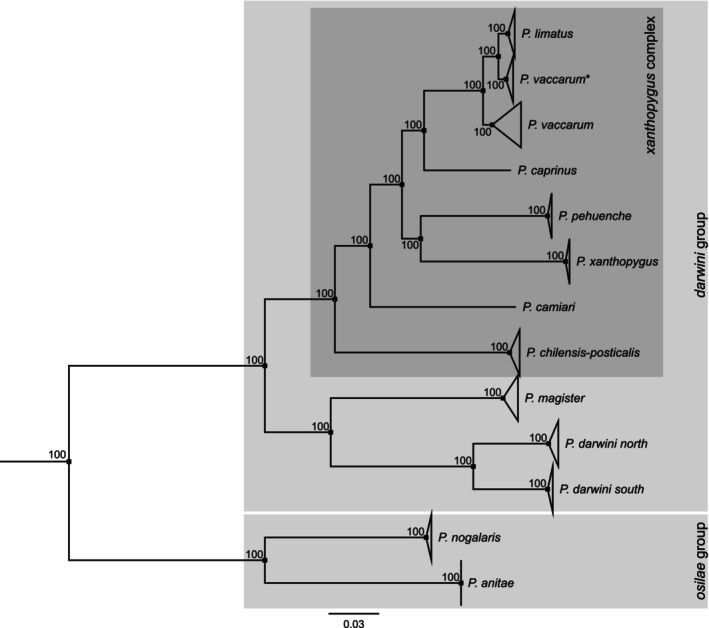
Maximum likelihood tree estimated from coding sequence of complete mitochondrial genomes for a set of 11 nominal *Phyllotis* species. Numbers adjacent to internal nodes denote ultrafast bootstrap support values for each clade. Within the taxon currently recognized as *P. darwini* , the species delimitation analysis identified two highly distinct subdivisions (see Figure 3). Representatives of both internal subdivisions form distinct clades in the mitogenome tree, which we labeled “ *P. darwini* south” and “ *P. darwini* ” north.

To further examine species limits suggested by the analysis of *cytb* sequence variation, we generated low‐coverage WGS data for representative subsets of specimens from 11 nominal species, several of which have overlapping ranges in the Altiplano and/or adjoining lowlands. We also derived an alignment of whole mitochondrial genomes from the WGS data. Whereas the BI and ML analyses of *cytb* variation yielded some conflicting estimates of species relationships within the *Phyllotis xanthopygus* species complex (Figure 2, Figure S1), the ML phylogeny estimate based on complete mitochondrial genomes placed *P. pehuenche* and *P. xanthopygus* sister to one another, consistent with the BI analysis of *cytb* sequences (Figure 4).

**FIGURE 5:**
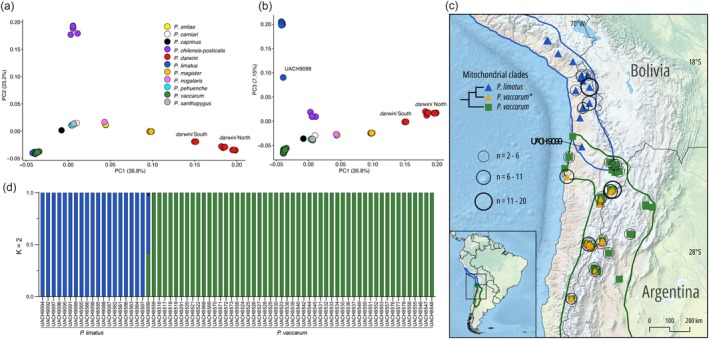
Genomic variation among species of *Phyllotis* based on 137 samples representing 11 nominal species. (a) Genomic principal component analysis (PCA) of genome‐wide variation (PC1 vs. PC2). Two distinct clusters of nominal *P. darwini* specimens, “*darwini* South” and “*darwini* North,” are distinguished along the PC1 axis. (b) Plot of PC1 vs. PC3 separates *P. limatus* and *P. vaccarum* along the PC3 axis, and reveals a single specimen, UACH9099 (designated *P. limatus* based on mtDNA haplotype), which has a PC3 score intermediate between the two species. (c) Map of collecting localities and distribution limits of *P. limatus* and *P. vaccarum*. UACH9099 comes from a site located in a narrow zone of range overlap between the two species in northern Chile. The map also shows the distribution of mice that are identified as *P. vaccarum* on the basis of whole‐genome sequence data, but which carry mtDNA haplotypes that are more closely related to those of *P. limatus* (denoted as “P. vaccarum*” in the inset tree diagram). (d) Structure plot showing clear distinction between *P. limatus* and *P. vaccarum* (*n* = 20 and 51, respectively). The putative hybrid specimen, UACH9099, was assigned almost exactly equal ancestry proportions from the two species.

In a PCA based on whole‐genome sequence variation, PC1, PC2, and PC3 captured 36.8%, 23.2%, and 7.15% of the total variation, respectively (Figure 5a,b). Samples of all species form distinct clusters in the multivariate space. Additionally, samples of *Phyllotis darwini* from the northern and southern portions of the species range separated into two highly distinct clusters (Figure 5a,b). The distinct clusters of *P. darwini* specimens identified in the genomic PCA are fully congruent with two divergent mtDNA clades that were identified as significant internal subdivisions in the species delimitation analysis (Figure 3). Similarly, the pairwise *p*‐distances estimated based on whole‐genome sequence variation are congruent with those *p*‐distances estimated based on *cytb* sequences (Tables S3 and S4). Using the coding sequence of the complete mitochondrial genome, the corresponding *p*‐distance between both clades of *P. darwini* was 7.25% (SE = 0.002).

**FIGURE 6:**
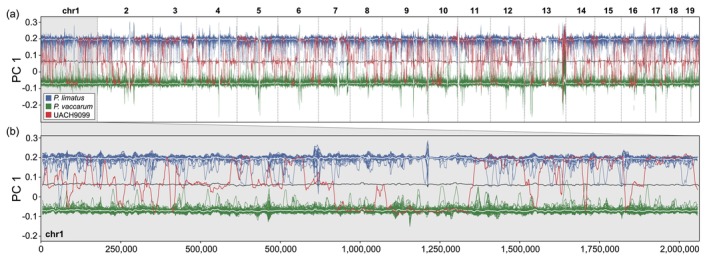
Windowed PCA of a *P. vaccarum* × *P. limatus* hybrid. PC1 was computed in overlapping 1 Mbp windows along the genome for a subset of 50 *P. vaccarum* (green), 20 *P. limatus* (blue), and the putative hybrid, UACH9099 (red). Mean PC1 values for each species are shown as white lines and the mean value between both species' averages is shown as a gray line. UACH9099 features a mosaic genome, with its local ancestry alternating between *P. vaccarum, P. limatus*, or a point intermediate between the two species. (a) Windowed PCA of chromosomes 1–19. (b) High resolution visualization of PC 1 along chromosome 1.

The sister species *Phyllotis limatus* and *P. vaccarum* were not readily distinguishable along the first two PC axes (Figure 5a), but they were cleanly separated along PC3 (Figure 5b). One specimen, UACH9099, which was identified as *P. limatus* on the basis of mtDNA, fell in between the two distinct clusters of *P. limatus* and *P. vaccarum* samples along PC3 (Figure 5b). Specimen UACH9099 was collected in the narrow zone of range overlap between southern *P. limatus* and northern *P. vaccarum*, about 200–250 km to the north of the localities where *P. vaccarum* specimens were found to carry mtDNA haplotypes more closely related to those of *P. limatus* than to other *P*. *vaccarum* (Figure 5c). While individual admixture proportions estimated with NGSadmix clearly distinguished *P. limatus* and *P. vaccarum* samples as genetically distinct clusters, specimen UACH9099 was assigned approximately equal admixture proportions of the two species (Figure 5d). A sliding window analysis of PC1 comprising the full sample of *P. limatus* and *P. vaccarum* specimens revealed a mosaic patterning of variation along the genome of UACH9099, as autosomal segments alternated between three main patterns: (i) homozygous for *P. limatus* ancestry, (ii) homozygous for *P. vaccarum* ancestry, or (iii) heterozygous, falling approximately halfway in between the two species (Figure 6).

### 3.3 Revised Geographic Range Limits of *Phyllotis* Species

**FIGURE 7:**
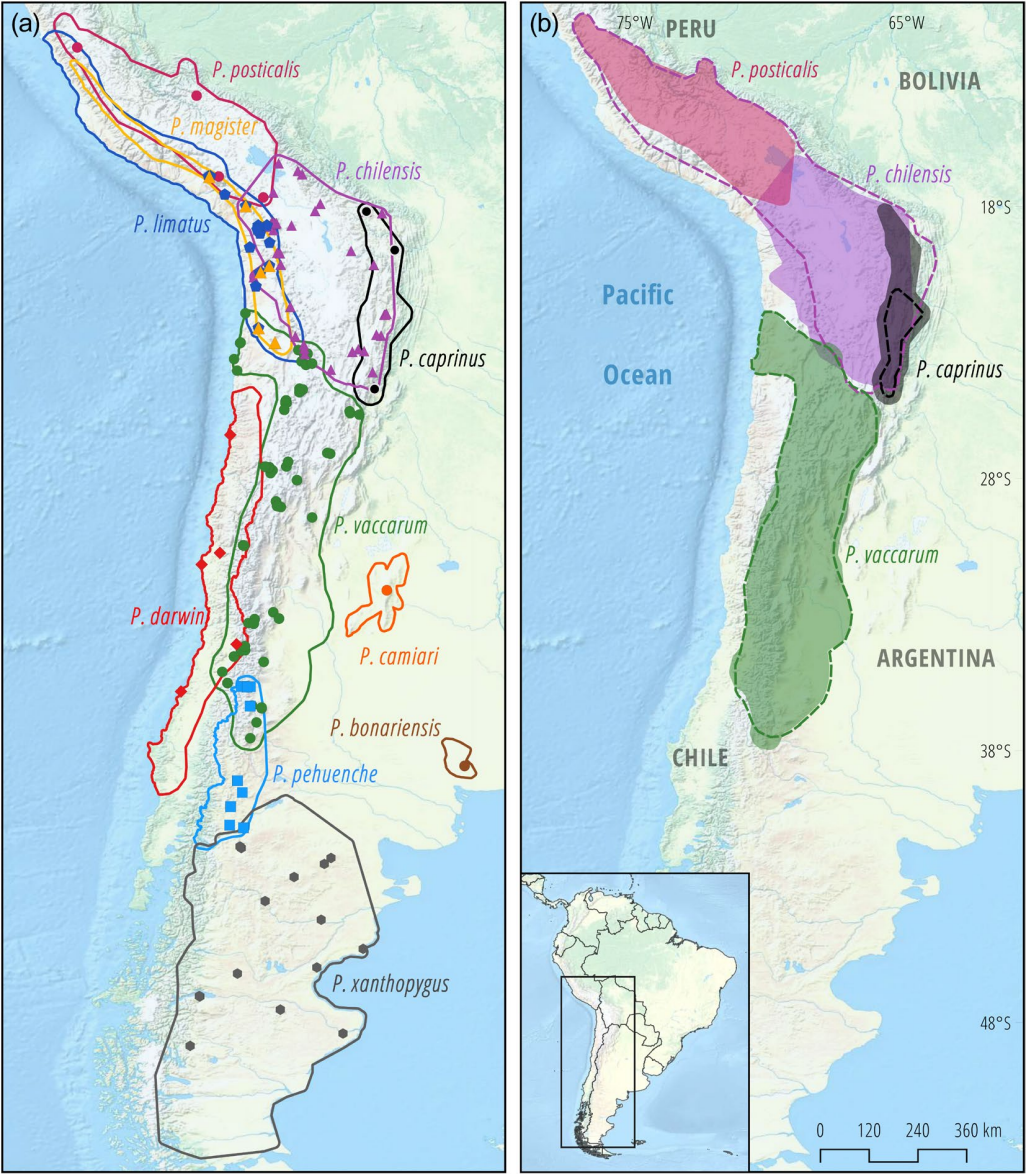
Revised distribution limits of *Phyllotis* species. (a) Inferred distributions of species in the *Phyllotis darwini* species group based on mtDNA and WGS data. Filled circles denote collection localities that helped define geographic range limits. (b) For several nominal species, sequence‐based identifications of newly collected specimens shifted known range limits (denoted by shading) relative to previously assumed limits (denoted by dashed lines).

The integrated analysis of mtDNA and WGS data enabled us to delineate the geographic range limits of several species in the surveyed region. The mice identified as *Phyllotis caprinus* that we collected in southern Bolivia significantly extend the known species range to the north (Figure 7). Another possibility suggested by the results of the species delimitation analysis (Figure 3) is that the specimens from central Bolivia do not represent extralimital records of *P. caprinus* but may instead represent an undescribed species sister to *P. caprinus s.s*. that occurs in southern Bolivia and northern Argentina. In the case of *P. chilensis* ‐*posticalis*, our specimens from the Chilean regions of Arica y Parinacota, Tarapacá, and Antofagasta extend the known species range to the west (Figure 7).

Our records of *Phyllotis vaccarum* indicate that this primarily highland species is replaced by *P. darwini* at elevations < 2000 m on the western slope of the Andes, but—beyond the northernmost limits of *P. darwini* —the range of *P*. *vaccarum* extends all the way to sea level along a narrow stretch of coastline in northern Chile (Figure 7). On the eastern slope of the Andes, our records from northwestern Argentina indicate that *P*. *vaccarum* does not occur below 1200 m, as it is replaced by *P. anitae* and *P. nogalaris* in lowland Yungas forests. Further south along the eastern slope of the Cordillera where humid lowland forests give way to arid steppe and Monte Desert habitats, our lowest elevation records of *P. vaccarum* were from 765–1158 m in the Argentine provinces of Catamarca, Mendoza, and Neuquén, but the majority of records are from elevations above 1200 m.

## 4 Discussion

### 4.1 Diversification of Current *Phyllotis* Species Occurred Mainly in the Pleistocene

Estimating divergence times of sigmodontine rodents has been difficult due to a lack of suitable fossils that could be used to calibrate phylogenetic trees (Parada et al. 2021). Previous studies, using a maximum likelihood clock estimate of 7.3% divergence per Mya (Steppan et al. 2004, 2007), placed the basal split of *Phyllotis* in the Pliocene (3.0–5.1 Mya) and the basal split of the *P. xanthopygus* species complex in the Pliocene–Pleistocene transition (1.6–2.3 Mya). Riverón (2011) estimated a similar Pliocene basal split for *Phyllotis* (2.83–4.05 Mya) using an analogous strict‐clock estimate. Our secondary calibration‐based inference suggests a similar timing of diversification of *Phyllotis*, with an estimated crown age of 4.51 Mya (95% HPD = 3.11–5.91 Mya) and subsequent diversification of the *P. xanthopygus* complex 2.78 Mya (95% HPD = 1.81–3.76 Mya). However, such inferences should be interpreted with caution since they are based on single locus data.

In principle, the diversification of *Phyllotis* could have been spurred by mountain uplift and/or climate‐related environmental changes at the end of the Pliocene and the beginning of the Pleistocene. The Central Andean Plateau is thought to have experienced the most significant phase of uplift during the late Miocene–Pliocene (Gregory‐Wodzicki 2000). The montane uplift hypothesis therefore predicts that diversification of *Phyllotis* would have started well before the end of the Pliocene (2.6 Mya). It is also possible that diversification occurred more recently and independently of Andean uplift during periods of climate‐induced environmental change in the Pleistocene. For example, the mid‐Pleistocene Transition (MPT; 1.25–0.70 Mya) was associated with a major shift in global climate periodicity that produced a persistent global aridification trend (Herbert 2023). Thus, the Pleistocene Aridification hypothesis predicts that major diversification of *Phyllotis* would have occurred more recently than the Andean uplift, coinciding with periods of climate change that were not directly related to orogenic events.

Our results suggest that most diversification of *Phyllotis* occurred during the past 3 million years, with divergence times for most species coinciding with glacial cycles in the mid‐ to late‐Pleistocene (Figure 2). Basal splits in two of the three main *Phyllotis* clades (the *andium‐amicus* and *osilae* species groups) occurred prior to the MPT (0.7–1.25 Mya), whereas the basal split within the *darwini* group is estimated to have occurred 2.78 Mya (95% HPD = 1.81–3.76 Mya), close to the Pliocene–Pleistocene boundary. Within each of the three main clades, most diversification events occurred within the past ~1.47–1.97 Mya. Thus, our results suggest that most diversification of *Phyllotis* would have occurred well after the late Miocene–Pliocene phase of Andean uplift.

### 4.2 Alpha Diversity Within the *Phyllotis darwini* Species Group

Based on results of the phylogenetic reconstructions and species delimitation analyses, we can identify at least 10 lineages that are referable to traditionally recognized forms within the *Phyllotis darwini* species group (Figures 2, 3, 4). However, results of the species delimitation analysis clearly show that some of these nominal forms may encompass more than one species. There appears to be potential for the existence of additional species within nominal forms that are currently recognized as *P. caprinus* , *P. chilensis‐posticalis*, *P. darwini* , *P. magister* , and *P*. *vaccarum* (Figure 3). The distinction of these candidate species requires further sampling and analysis, as results of species limitation approaches based on sequence data are best combined with other lines of evidence as a basis for taxonomic revision (Hillis et al. 2021; Sukumaran et al. 2021).

The Bolivian specimens of *Phyllotis caprinus* from Chuquisaca (MSB237236) and Cochabamba (MSB238568) constitute a highly differentiated clade relative to Argentine specimens that are referable to topotypical *P. caprinus* (*cytb p*‐distance = 5.6%, SE = 0.008) (Figure 3). Similarly, *Phyllotis darwini* and *P. chilensis‐posticalis* also exhibit north–south patterns of internal structure (Figure S2), with highly distinct units identified by the species delimitation analyses (Figure 3). In the case of *P. darwini* , divergence between northern and southern mtDNA clades is also apparent at the whole‐genome level (Figure 5a,b). Consistent with the results of Ojeda et al. (2021), the clade that includes specimens that we refer to as *P. chilensis‐posticalis* appears likely to contain multiple species with apparently allopatric distributions (Figure S2b). Although Ojeda et al. (2021) referred to this group as to “*P. posticalis‐rupestris*,” geographic considerations of type localities suggest that *P. chilensis‐posticalis* is more appropriate as a provisional name for the subclade with the southern‐most distribution in northeastern Chile, southwestern Bolivia, and northwestern Argentina (D'Elía et al. in preparation; Figure S2b). Here and elsewhere (Storz et al. 2024) we followed Mann (1945) and Pearson (1958) in using the name *P. chilensis* for the mice in the subclade that we collected in the Altiplano of northern Chile, southwestern Bolivia, and northwestern Argentina. Therefore, we prioritize the use of *P. posticalis* for the subclade with the northern‐most distribution as it includes a specimen from the vicinity of the type locality of *posticalis* in the Department of Junín, Peru (Thomas 1912). Assessment of possible species‐level distinctions between the two main lineages of *P. chilensis‐posticalis* (Figure 3) requires further analyses using morphological and genomic data, ideally including an evaluation of the corresponding holotype specimens. It is worth noting that all four sequenced specimens from Parinacota, Región de Arica y Parinacota (the type locality of both *P. chilensis* and *P. osgoodi* ) carry mtDNA haplotypes that fall in the clade of *P. limatus* . Broader specimen collection and character sampling is needed to clarify whether more than one *Phyllotis* species exists in Parinacota and to explore the possibility that *P. chilensis* and/or *P. osgoodi* is a synonym of *P. limatus* .

In *Phyllotis vaccarum*, one *cytb* haplogroup that was identified as a distinct unit in the species delimitation analysis is sister to a clade formed by haplotypes of *P. limatus* . However, the specimens of *P. vaccarum* that harbor mtDNA more closely related to that of *P. limatus* than to other *P. vaccarum* are not distinguishable from other *P. vaccarum* at the whole‐genome level (Storz et al. 2024). In this case of mitonuclear discordance, identified mtDNA subdivisions are clearly not reflective of cryptic species within *P. vaccarum*.

### 4.3 Evidence for Interspecific Hybridization

The genomic data revealed clear‐cut evidence of ongoing hybridization between *Phyllotis limatus* and *P. vaccarum* (Figures 5d and 6), suggesting that introgression is a plausible explanation for the observed mitonuclear discordance between the two species (Figure 5c; Storz et al. 2024). Specimen UACH9099, which carries mtDNA of *P. limatus* , harbors approximately equal genome‐wide admixture proportions from *P. limatus* and *P. vaccarum* (Figure 5d). At face value, the approximately equal admixture proportions seemingly suggest that UACH9099 could be a first generation (F1) interspecific hybrid that received one haploid complement of chromosomes from each parental species. However, in the windowed PCA, an F1 hybrid would be expected to localize halfway between the two divergent parental stocks. Contrary to that expectation, tracts across the genome of UACH9099 were either homozygous for *P. vaccarum* ancestry, homozygous for *P. limatus* ancestry, or heterozygous (i.e., combining genomes of both species) (Figure 6). The mosaic patterning of nucleotide variation therefore indicates that one or more rounds of recombination occurred subsequent to an initial *P. limatus* × *P. vaccarum* hybridization event, revealing that UACH9099 must be the product of an F2 or more advanced‐stage intercross.

Given that UACH9099 was assigned roughly equal admixture proportions for both species (Figure 5c), it is likely that the zone of range overlap between *P. limatus* and *P. vaccarum* in northern Chile represents a zone of ongoing hybridization. Although the observed pattern of genomic mosaicism in UACH9099 could have been produced by a balanced number of backcrossing events with both parental species, we regard ongoing matings between hybrids as a more likely scenario. More intensive collecting from the zone of range overlap between *P. limatus* and *P. vaccarum* will be required to assess the pervasiveness of hybridization between the two species. A similar pattern of mitonuclear discordance was documented in another pair of closely related phyllotine species in the genus *Eligmodontia*, and was interpreted as a result of asymmetric introgression (Armella Sierra et al. 2017).

Aside from the evidence of hybridization and mitonuclear discordance between *Phyllotis limatus* and *P. vaccarum*, which also happen to be the only pair of sister species with overlapping ranges within the *P. darwini* group, all remaining *Phyllotis* specimens that grouped together in the *cytb* phylogeny were also identified as distinct groupings in the analysis of WGS data (Figure 5a,b).

### 4.4 A Revised Understanding of Geographical Range Limits of *Phyllotis* Mice

The use of sequence data to confirm the identities of all collected specimens provided new information about geographic range limits and revealed notable range extensions for several species of *Phyllotis* (Figure 7). The westward range extension of *Phyllotis chilensis* in northern Chile is noteworthy because only *P. limatus* and *P. magister* had been previously recorded in this zone (Steppan and Ramírez 2015; Ojeda et al. 2021). We collected specimens referable to *P. chilensis* from several extremely high‐elevation localities in northern Chile and western Bolivia, including multiple specimens from 5221 m on the flanks of Volcán Parinacota and 5027 m on the flanks of Volcán Acotango in western Bolivia. Such records highlight the importance of surveying environmental extremes to accurately characterize geographic range limits, especially for taxa like *Phyllotis* that are known to inhabit extreme southern latitudes in Patagonia, extreme elevations in the Central Andes, and extreme arid zones in the Atacama Desert. *P. vaccarum* was previously documented to have the broadest elevational range of any mammal, from the coastal desert of northern Chile to the summits of > 6700 m volcanoes (Storz et al. 2020, 2024). The species has a similarly broad elevational range on the eastern slope of the Andes, but the lower range limit depends on the nature of the low elevation biome (Jayat et al. 2021; Riverón 2011). In northwest Argentina, the species appears to have a lower range limit > 1200 m, as it is replaced by species in the *osilae* group in humid Yungas forests. In central western Argentina, *P. vaccarum* reaches elevations below 1000 m in arid Patagonian steppe and Monte habitats.

## 5 Conclusions

Our intensive collecting of *Phyllotis* in the Andean highlands and surrounding lowlands enabled us to fill key gaps in geographic coverage. By integrating vouchered specimen records with species identifications based on phenotypic, mtDNA, and WGS data, we now have a better understanding of geographic range limits for species of the *darwini* species group. The delimitation of genetically distinct units within several recognized species indicates the likely presence of much undescribed alpha diversity in *Phyllotis*, as pointed out by previous authors (e.g., Ojeda et al. 2021; Jayat et al. 2021). Within the *P. xanthopygus* species complex, *P. limatus* and *P. vaccarum* represent the only species for which we observed mitonuclear discordance and documented ongoing hybridization. This example indicates that interspecific hybridization occurs in *Phyllotis*, but more intensive collecting in zones of range overlap between species will be required to assess the pervasiveness of introgressive hybridization in the group. Although much of the diversification of *Phyllotis* may have occurred in the Andean highlands, our divergence date estimates suggest that most diversification of these mice was not associated with the major phase of uplift of the Central Andean Plateau in the Miocene–late Pliocene. Instead, most lineage splitting seems to be associated with climatically induced environmental changes in the mid‐ to late‐Pleistocene.

## Author Contributions

**Marcial Quiroga‐Carmona:** conceptualization (equal), data curation (equal), formal analysis (equal), investigation (equal), methodology (equal), resources (equal), software (equal), supervision (equal), validation (equal), visualization (equal), writing – original draft (equal), writing – review and editing (equal). **Schuyler Liphardt:** data curation (equal), formal analysis (equal), investigation (equal), methodology (equal), software (equal), writing – review and editing (equal). **Naim M. Bautista:** validation (equal), visualization (equal), writing – review and editing (equal). **Pablo Jayat:** data curation (equal), validation (equal), writing – review and editing (equal). **Pablo Teta:** data curation (equal), validation (equal), writing – review and editing (equal). **Jason L. Malaney:** data curation (equal), validation (equal), writing – review and editing (equal). **Tabitha McFarland:** data curation (equal), validation (equal), writing – review and editing (equal). **Joseph A. Cook:** data curation (equal), validation (equal), writing – review and editing (equal). **L. Moritz Blumer:** methodology (equal), software (equal), visualization (equal), writing – review and editing (equal). **Nathanael D. Herrera:** data curation (equal), formal analysis (equal), methodology (equal), validation (equal), writing – review and editing (equal). **Zachary A. Cheviron:** validation (equal), writing – review and editing (equal). **Jeffrey M. Good:** validation (equal), writing – review and editing (equal). **Guillermo D'Elía:** conceptualization (equal), funding acquisition (equal), investigation (equal), methodology (equal), supervision (equal), validation (equal), writing – review and editing (equal). **Jay F. Storz:** conceptualization (equal), funding acquisition (equal), investigation (equal), methodology (equal), project administration (equal), supervision (equal), validation (equal), writing – original draft (equal), writing – review and editing (equal).

## Ethics Statement

All animals were collected in the field with permission from the following agencies: Servicio Agrícola y Ganadero, Chile (6633/2020, 2373/2021, 5799/2021, 3204/2022, 3565/2022, 911/2023 and 7736/2023), Corporación Nacional Forestal, Chile (171219, 1501221, and 31362839), Dirección Nacional de Fronteras y Límites del Estado, Chile (DIFROL, Autorización de Expedición Científica #68 and 02/22), Ministerio de Medio Ambiente y Agua, Estado Plurinacional de Bolivia (Resolución Administrativa 026/09 and DVS‐CRT‐02/91), and the Secretaria de Ambiente (Ministerio de Produccion y Ambiente) de La Rioja, the Ministerio de Ambiente (Secretaria de Biodiversidad y Desarrollo Sustentable) de Jujuy, and the Ministerio de Desarrollo Productivo (Direccion de Flora, Fauna Silvestre y Suelos) de Tucumán, Argentina (Expte. N° P4‐00402‐21 Disp. S.A. N° 001/22, Expte. N° P4‐00158‐22 Disp. S.A. N° 007/22, Expte. N° 677‐330‐2021, and Expte. N° 677‐330‐2021). All live‐trapped animals were handled in accordance with protocols approved by the Institutional Animal Care and Use Committee (IACUC) of the University of Nebraska (project ID's: 1919, 2100), IACUC of the University of New Mexico (project ID's: 16787 and 20405), and the bioethics committee of the Universidad Austral de Chile (certificate 456/2022).

## Conflicts of Interest

The authors declare no conflicts of interest.

## Supporting information


Appendix S1.

## Data Availability

The genomic data associated with this study are openly available in the NCBI bioproject PRJNA950396. The newly generated *cytb* sequences are available in GenBank (accession numbers: PQ295377–PQ295555).

## References

[ece371783-bib-0001] Aktas, C. 2023. “Haplotypes: Manipulating DNA Sequences and Estimating Unambiguous Haplotype Network With Statistical Parsimony.” R Package Version 1.1.3.1. https://CRAN.R‐project.org/package=haplotypes.

[ece371783-bib-0002] Anderson, S. , and T. L. Yates . 2000. “A New Genus and Species of Phyllotine Rodent From Bolivia.” Journal of Mammalogy 81: 18–36.

[ece371783-bib-0003] Armella Sierra, A. B. , E. R. Castillo , C. Labaroni , et al. 2017. “Genetic Studies in the Recently Divergent *Eligmodontia puerulus* and *E. moreni* (Rodentia, Cricetidae, Sigmodontinae) From Puna and Monte Deserts of South America.” Mammalian Biology 87: 93–100.

[ece371783-bib-0004] Bouckaert, R. , J. Heled , D. Kühnert , et al. 2014. “BEAST 2: A Software Platform for Bayesian Evolutionary Analysis.” PLoS Computational Biology 10: e1003537. 24722319 10.1371/journal.pcbi.1003537 PMC3985171

[ece371783-bib-0005] Cadenillas, R. , and G. D'Elía . 2021. “Taxonomic Revision of the Populations Assigned to *Octodon degus* (Hystricomorpha: Octodontidae): With the Designation of a Neotype for *Sciurus Degus* G. I. Molina, 1782 and the Description of a New Subspecies.” Zoologischer Anzeiger 292: 14–28.

[ece371783-bib-0006] Chen, S. , Y. Zhou , Y. Chen , and J. Gu . 2018. “fastp: An Ultra‐Fast All‐In‐One FASTQ Preprocessor.” Bioinformatics 34: i884–i890. 30423086 10.1093/bioinformatics/bty560 PMC6129281

[ece371783-bib-0007] Dellicour, S. , and J. F. Flot . 2018. “The Hitchhiker's Guide to Single‐Locus Species Delimitation.” Molecular Ecology Resources 18: 1234–1246. 29847023 10.1111/1755-0998.12908

[ece371783-bib-0008] Dierckxsens, N. , P. Mardulyn , and G. Smits . 2017. “NOVOPlasty: de Novo Assembly of Organelle Genomes From Whole Genome Data.” Nucleic Acids Research 45: e18. 28204566 10.1093/nar/gkw955 PMC5389512

[ece371783-bib-0009] Fujisawa, T. , and T. G. Barraclough . 2013. “Delimiting Species Using Single‐Locus Data and the Generalized Mixed Yule Coalescent Approach: A Revised Method and Evaluation on Simulated Data Sets.” Systematic Biology 62: 707–724. 23681854 10.1093/sysbio/syt033 PMC3739884

[ece371783-bib-0010] Gregory‐Wodzicki, K. M. 2000. “Uplift History of the Central and Northern Andes: A Review.” Geological Society of America Bulletin 112: 1091–1105.

[ece371783-bib-0011] Herbert, T. D. 2023. “The Mid‐Pleistocene Climate Transition.” Annual Review of Earth and Planetary Sciences 51: 389–418.

[ece371783-bib-0012] Hershkovitz, P. 1962. “Evolution of Neotropical Cricetine Rodents (Muridae) With Special Reference to the Phyllotine Group.” Fieldiana Zoology 46: 1–524.

[ece371783-bib-0013] Hillis, D. M. , E. A. Chambers , and T. J. Devitt . 2021. “Contemporary Methods and Evidence for Species Delimitation.” Ichthyology & Herpetology 109, no. 3: 895–903.

[ece371783-bib-0014] Jayat, J. P. , G. D'Elía , U. F. J. Pardiñas , and J. G. Namen . 2007. “A New Species of *Phyllotis* (Rodentia, Cricetidae, Sigmodontine) From the Upper Montane Forest of the Yungas of North‐Western Argentina.” In The Quintessential Naturalist: Honoring the Life and Legacy of Oliver P. Pearson, edited by D. A. Kelt , E. P. Lessa , J. Salazar‐Bravo , and J. L. Patton , 775–798. University of California. Publications in Zoology, 134.

[ece371783-bib-0015] Jayat, J. P. , P. E. Ortiz , F. R. González , and G. D'Elía . 2016. “Taxonomy of the *Phyllotis osilae* Species Group in Argentina; the Status of the “Rata de los Nogales” (*Phyllotis nogalaris* Thomas, 1921; Rodentia: Cricetidae).” Zootaxa 4083: 397–417. 27394238 10.11646/zootaxa.4083.3.5

[ece371783-bib-0016] Jayat, J. P. , P. Teta , A. A. Ojeda , et al. 2021. “The *Phyllotis xanthopygus* Complex (Rodentia, Cricetidae) in Central Andes, Systematics and Description of a New Species.” Zoologica Scripta 50: 689–706.

[ece371783-bib-0017] Kalyaanamoorthy, S. , B. Q. Minh , T. K. Wong , A. von Haeseler , and L. S. Jermiin . 2017. “ModelFinder: Fast Model Selection for Accurate Phylogenetic Estimates.” Nature Methods 14: 587–589. 28481363 10.1038/nmeth.4285 PMC5453245

[ece371783-bib-0018] Katoh, K. , J. Rozewicki , and K. D. Yamada . 2017. “MAFFT Online Service: Multiple Sequence Alignment, Interactive Sequence Choice and Visualization.” Briefings in Bioinformatics 20: 1160–1166. 10.1093/bib/bbx108 PMC6781576 28968734

[ece371783-bib-0019] Katoh, K. , and D. M. Standley . 2013. “MAFFT Multiple Sequence Alignment Software Version 7: Improvements in Performance and Usability.” Molecular Biology and Evolution 30: 772–780. 23329690 10.1093/molbev/mst010 PMC3603318

[ece371783-bib-0020] Korneliussen, T. S. , A. Albrechtsen , and R. Nielsen . 2014. “ANGSD: Analysis of Next Generation Sequencing Data.” BMC Bioinformatics 15: 356. 25420514 10.1186/s12859-014-0356-4 PMC4248462

[ece371783-bib-0021] Kumar, S. , G. Stecher , M. Li , C. Knyaz , and K. Tamura . 2018. “MEGA X: Molecular Evolutionary Genetics Analysis Across Computing Platforms.” Molecular Biology and Evolution 35: 1547–1549. 29722887 10.1093/molbev/msy096 PMC5967553

[ece371783-bib-0022] Larsson, A. 2014. “AliView: A Fast and Lightweight Alignment Viewer and Editor for Large Data Sets.” Bioinformatics 30: 3276–3278. 25095880 10.1093/bioinformatics/btu531 PMC4221126

[ece371783-bib-0023] Li, H. , and R. Durbin . 2009. “Fast and Accurate Short Read Alignment With Burrows‐Wheeler Transform.” Bioinformatics 25: 1754–1760. 19451168 10.1093/bioinformatics/btp324 PMC2705234

[ece371783-bib-0024] Li, H. , B. Handsaker , A. Wysoker , et al. 2009. “The Sequence Alignment/Map Format and SAMtools.” Bioinformatics 25: 2078–2079. 19505943 10.1093/bioinformatics/btp352 PMC2723002

[ece371783-bib-0025] Mammal Diversity Database . 2025. “Mammal Diversity Database (v2.1) [Data Set].” *Zenodo*. 10.5281/zenodo.15163494.

[ece371783-bib-0074] Mann, G. 1945. “Mamíferos de Tarapacá. Observaciones Realizadas Durante una Expedición al Alto Norte de Chile.” Biológica 2: 23–134.

[ece371783-bib-0026] McKenna, A. , M. Hanna , E. Banks , et al. 2010. “The Genome Analysis Toolkit: A MapReduce Framework for Analyzing Next‐Generation DNA Sequencing Data.” Genome Research 20: 1297–1303. 20644199 10.1101/gr.107524.110 PMC2928508

[ece371783-bib-0027] Meisner, J. , and A. Albrechtsen . 2018. “Inferring Population Structure and Admixture Proportions in Low‐Depth NGS Data.” Genetics 210: 719–731. 30131346 10.1534/genetics.118.301336 PMC6216594

[ece371783-bib-0028] Minh, B. Q. , M. A. Thi Nguyen , and A. von Haeseler . 2013. “Ultrafast Approximation for Phylogenetic Bootstrap.” Molecular Biology and Evolution 30: 1188–1195. 23418397 10.1093/molbev/mst024 PMC3670741

[ece371783-bib-0029] Novillo, A. , C. Lanzone , J. P. Jayat , et al. 2024. “Beta Diversity Patterns in Andean Rodents: Current and Historical Factors as Drivers of Turnover and Nestedness.” Journal of Mammalogy 105: 230–240.

[ece371783-bib-0030] Ojeda, A. A. , P. Teta , J. P. Jayat , et al. 2021. “Phylogenetic Relationships Among Cryptic Species of the *Phyllotis xanthopygus* Complex (Rodentia, Cricetidae).” Zoologica Scripta 50: 269–281.

[ece371783-bib-0031] Pajares, A. J. M. 2013. “SIDIER: Substitution and Indel Distances to Infer Evolutionary Relationships.” Methods in Ecology and Evolution 4: 1195–1200.

[ece371783-bib-0032] Parada, A. , G. D'Elía , and R. E. Palma . 2015. “The Influence of Ecological and Geographical Context in the Radiation of Neotropical Sigmodontine Rodents.” BMC Evolutionary Biology 15: 172. 26307442 10.1186/s12862-015-0440-z PMC4549906

[ece371783-bib-0033] Parada, A. , J. Hanson , and G. D'Eiía . 2021. “Ultraconserved Elements Improve the Resolution of Difficult Nodes Within the Rapid Radiation of Neotropical Sigmodontine Rodents (Cricetidae: Sigmodontinae).” Systematic Biology 70: 1090–1100. 33787920 10.1093/sysbio/syab023

[ece371783-bib-0034] Parada, A. , U. F. Pardiñas , J. Salazar‐Bravo , G. D'Elía , and R. E. Palma . 2013. “Dating an Impressive Neotropical Radiation: Molecular Time Estimates for the Sigmodontinae (Rodentia) Provide Insights Into Its Historical Biogeography.” Molecular Phylogenetics and Evolution 66: 960–968. 23257216 10.1016/j.ympev.2012.12.001

[ece371783-bib-0035] Pardiñas, U. F. J. , G. D'Elía , and P. E. Ortiz . 2002. “Sigmodontinos fósiles (Rodentia, Muroidea, Sigmodontinae) de América del Sur: estado actual de su conocimiento y prospectiva.” Mastozoología Neotropical 9: 209–252.

[ece371783-bib-0037] Pearson, O. P. 1958. “A Taxonomic Revision of the Rodent Genus *Phyllotis* .” University of California Publications in Zoology 56: 391–496.

[ece371783-bib-0038] Pearson, O. P. , and J. L. Patton . 1976. “Relationships Among South American Phyllotine Rodents Based on Chromosome Analysis.” Journal of Mammalogy 57: 339–350.

[ece371783-bib-0039] Pons, J. , T. G. Barraclough , J. Gómez‐Zurita , et al. 2006. “Sequence‐Based Species Delimitation for the DNA Taxonomy of Undescribed Insects.” Systematic Biology 55: 595–609. 16967577 10.1080/10635150600852011

[ece371783-bib-0040] Quiroga‐Carmona, M. , J. F. Storz , and G. D'Elía . 2023. “Elevational Range Extension of the Puna Mouse, *Punomys* (Cricetidae), With the First Record of the Genus From Chile.” Journal of Mammalogy 104: 1144–1151. 37800100 10.1093/jmammal/gyad064 PMC10550245

[ece371783-bib-0041] R Core Team . 2020. R: A Language and Environment for Statistical Computing. R Foundation for Statistical Computing.

[ece371783-bib-0042] Rambaut, A. , and A. J. Drummond . 2019. TreeAnnotator v2 6.0–MCMC Output Analysis. Software Development. Part of Beast, 2.

[ece371783-bib-0043] Rambaut, A. , A. J. Drummond , D. Xie , G. Baele , and M. A. Suchard . 2018. “Posterior Summarization in Bayesian Phylogenetics Using Tracer 1.7.” Systematic Biology 67: 901–904. 29718447 10.1093/sysbio/syy032 PMC6101584

[ece371783-bib-0044] Reid, N. M. , and B. C. Carstens . 2012. “Phylogenetic Estimation Error Can Decrease the Accuracy of Species Delimitation: A Bayesian Implementation of the General Mixed Yule‐Coalescent Model.” BMC Evolutionary Biology 12: 196. 23031350 10.1186/1471-2148-12-196 PMC3503838

[ece371783-bib-0046] Rengifo, E. M. , and V. Pacheco . 2015. “Taxonomic Revision of the Andean Leaf–Eared Mouse, *Phyllotis andium* Thomas 1912 (Rodentia: Cricetidae), With the Description of a New Species.” Zootaxa 4018: 349–380. 26624045 10.11646/zootaxa.4018.3.2

[ece371783-bib-0047] Rengifo, E. M. , and V. Pacheco . 2017. “Phylogenetic Position of the Ancash Leaf‐Eared Mouse *Phyllotis definitus* Osgood 1915 (Rodentia: Cricetidae).” Mammalia 82: 153–166.

[ece371783-bib-0048] Riverón, S. 2011. “Estructura poblacional e historia demográfica del “pericote patagónico” *Phyllotis xanthopygus* (Rodentia: Sigmodontinae) en Patagonia Argentina.” PhD diss., Universidad de la República, pp. 100.

[ece371783-bib-0049] Salazar‐Bravo, J. , U. F. Pardiñas , and G. D'Elía . 2013. “A Phylogenetic Appraisal of Sigmodontinae (Rodentia, Cricetidae) With Emphasis on Phyllotine Genera: Systematics and Biogeography.” Zoologica Scripta 42: 250–261.

[ece371783-bib-0051] Skotte, L. , T. S. Korneliussen , and A. Albrechtsen . 2013. “Estimating Individual Admixture Proportions From Next Generation Sequencing Data.” Genetics 195, no. 3: 693–702. 24026093 10.1534/genetics.113.154138 PMC3813857

[ece371783-bib-0052] Smith, M. F. , and J. L. Patton . 1993. “The Diversification of South American Murid Rodents: Evidence From Mitochondrial DNA Sequence Data for the Akodontine Tribe.” Biological Journal of the Linnean Society 50: 149–177.

[ece371783-bib-0053] Steppan, S. J. 1993. “Phylogenetic Relationships Among the Phyllotini (Rodentia: Sigmodontinae) Using Morphological Characters.” Journal of Mammalian Evolution 1: 187–213.

[ece371783-bib-0054] Steppan, S. J. 1995. Revision of the Tribe Phyllotini (Rodentia: Sigmodontinae), With a Phylogenetic Hypothesis for the Sigmodontinae. Vol. 80, 1–112. Fieldiana Zoology.

[ece371783-bib-0055] Steppan, S. J. 1998. “Phylogenetic Relationships and Species Limits Within *Phyllotis* (Rodentia: Sigmodontinae): Concordance Between mtDNA Sequence and Morphology.” Journal of Mammalogy 79: 573–593.

[ece371783-bib-0056] Steppan, S. J. , R. M. Adkins , and J. Anderson . 2004. “Phylogeny and Divergence‐Date Estimates of Rapid Radiations in Muroid Rodents Based on Multiple Nuclear Genes.” Systematic Biology 53, no. 4: 533–553. 15371245 10.1080/10635150490468701

[ece371783-bib-0057] Steppan, S. J. , T. Bowen , M. R. Bangs , et al. 2022. “Evidence of a Population of Leaf‐Eared Mice (*Phyllotis Vaccarum*) Above 6000 m in the Andes and a Survey of High‐Elevation Mammals.” Journal of Mammalogy 103: 776–785. 36118797 10.1093/jmammal/gyac028 PMC9469927

[ece371783-bib-0058] Steppan, S. J. , and O. Ramírez . 2015. “Genus *Phyllotis* Waterhouse, 1837.” In Mammals of South America, Volume 2, Rodents, edited by J. L. Patton , U. F. J. Pardiñas , and G. D'Elía , 535–555. University of Chicago Press.

[ece371783-bib-0059] Steppan, S. J. , O. Ramírez , J. Banbury , et al. 2007. “A Molecular Reappraisal of the Systematics of the Leaf–Eared Mice *Phyllotis* and Their Relatives.” In The Quintessential Naturalist: Honoring the Life and Legacy of Oliver Pearson, edited by D. A. Kelt , E. P. Lessa , J. Salazar‐Bravo , and J. L. Patton , 799–826. University of California, Publications of Zoology.

[ece371783-bib-0061] Storz, J. F. , S. Liphardt , M. Quiroga‐Carmona , et al. 2023. “Genomic Insights Into the Mystery of Mouse Mummies on the Summits of Atacama Volcanoes.” Current Biology 33: R1040–R1042. 37875074 10.1016/j.cub.2023.08.081 PMC10652914

[ece371783-bib-0062] Storz, J. F. , M. Quiroga‐Carmona , S. Liphardt , et al. 2024. “Extreme High‐Elevation Mammal Surveys Reveal Unexpectedly High Upper Range Limits of Andean Mice.” American Naturalist 203, no. 6: 726–735. 10.1086/729513 PMC12232203 38781524

[ece371783-bib-0063] Storz, J. F. , M. Quiroga‐Carmona , J. C. Opazo , et al. 2020. “Discovery of the World's Highest‐Dwelling Mammal.” Proceedings of the National Academy of Sciences of the United States of America 117, no. 18: 18,169–18,171. 32675238 10.1073/pnas.2005265117 PMC7414144

[ece371783-bib-0064] Storz, J. F. , and G. R. Scott . 2024. “To What Extent Do Physiological Tolerances Determine Elevational Range Limits of Mammals?” Journal of Physiology 602: 5475–5484. 37889163 10.1113/JP284586 PMC11052920

[ece371783-bib-0065] Sukumaran, J. , M. T. Holder , and L. L. Knowles . 2021. “Incorporating the Speciation Process Into Species Delimitation.” PLoS Computational Biology 17, no. 5: e1008924. 33983918 10.1371/journal.pcbi.1008924 PMC8118268

[ece371783-bib-0066] Teta, P. , J. P. Jayat , C. Lanzone , A. Novillo , A. Ojeda , and R. A. Ojeda . 2018. “Geographic Variation in Quantitative Skull Traits and Systematics of Southern Populations of the Leaf‐Eared Mice of the *Phyllotis xanthopygus* Complex (Cricetidae, Phyllotini) in Southern South America.” Zootaxa 4446: 68–80. 30313897 10.11646/zootaxa.4446.1.5

[ece371783-bib-0067] Teta, P. , J. P. Jayat , S. J. Steppan , et al. 2022. “Uncovering Cryptic Diversity Does Not End: A New Species of Leaf‐Eared Mouse, Genus *Phyllotis* (Rodentia, Cricetidae), From Central Sierras of Argentina.” Mammalia 86, no. 4: 393–405.

[ece371783-bib-0068] Thomas, O. 1912. “New Bats and Rodents From South America.” Annals and Magazine of Natural History 8, no. 10: 403–411.

[ece371783-bib-0069] Trifinopoulos, J. , L. T. Nguyen , A. von Haeseler , and B. Q. Minh . 2016. “W‐IQ‐TREE: A Fast Online Phylogenetic Tool for Maximum Likelihood Analysis.” Nucleic Acids Research 44: W232–W235. 27084950 10.1093/nar/gkw256 PMC4987875

[ece371783-bib-0070] Vieira, F. G. , F. Lassalle , T. S. Korneliussen , and M. Fumagalli . 2015. “Improving the Estimation of Genetic Distance From Next‐Generation Sequencing Data.” Biological Journal of the Linnean Society 117, no. 1: 139–149.

[ece371783-bib-0071] Walker, L. I. , A. E. Spotorno , and J. Arrau . 1984. “Cytogenetic and Reproductive Studies of Two Nominal Subspecies of *Phyllotis darwini* and Their Experimental Hybrids.” Journal of Mammalogy 65: 220–230.

[ece371783-bib-0072] Wickham, H. 2016. ggplot2: Elegant Graphics for Data Analysis. 2nd ed. Springer.

[ece371783-bib-0073] Zhang, J. , P. Kapli , P. Pavlidis , and A. Stamatakis . 2013. “A General Species Delimitation Method With Applications to Phylogenetic Placements.” Bioinformatics 29, no. 22: 2869–2876. 23990417 10.1093/bioinformatics/btt499 PMC3810850

