# Supplementary material for: Species Limits and Hybridization in Andean Leaf‐Eared Mice (Phyllotis)
Source: Ecol Evol. 2025 Jul 13;15(7):e71783. doi: 10.1002/ece3.71783 (PMC12256280; doi:10.1002/ece3.71783)
Supplement: Supplementary file 1 — Appendix S1. [file ECE3-15-e71783-s001.pdf]

TABLE S1. Specimens of the genus *Phyllotis* included in this study. Information for each specimen includes *cytb* haplotype class (cf. Figure 3), voucher and field number, GenBank accession number, country, and locality description.

Voucher number	Field number	GenBank	Species	Haplotype class	Country	Locality
CMI7317	AN13	MT776505	Phyllotis vaccarum	Hap_004	Argentina	Puente del Inca
	AN17	MT776485	Phyllotis pehuenche	Hap_005	Argentina	3 km NW from Las Leñas
CMI7324	AN53	MT776480	Phyllotis pehuenche	Hap_016	Argentina	Laguna de la Niña Encantada
	AN69	MT776507	Phyllotis vaccarum	Hap_006	Argentina	31.7 km west from Uspallata
CMI7410	AN70	MT776503	Phyllotis vaccarum	Hap_007	Argentina	31.7 km west from Uspallata
CMI7398	AN74	MT776501	Phyllotis vaccarum	Hap_008	Argentina	A 68 km de Horcones, 2,854 m
	AN75	MT776500	Phyllotis vaccarum	Hap_008	Argentina	A 68 km de Horcones, 2,854 m
	AN76	MT776506	Phyllotis vaccarum	Hap_009	Argentina	Mendoza, 31 km west from Uspallata
	AN84	MT776478	Phyllotis pehuenche	Hap_017	Argentina	Arroyo El Seguro
CMI7421	AN85	MT776479	Phyllotis pehuenche	Hap_018	Argentina	Arroyo El Seguro
	AO79	MT776494	Phyllotis vaccarum	Hap_010	Argentina	Pampa Palauco, Yacimiento YPF
	AO82	MT776493	Phyllotis vaccarum	Hap_010	Argentina	Pampa Palauco, Cajon de Letellier
CMI7505	AO87	MT776495	Phyllotis vaccarum	Hap_010	Argentina	Pampa Palauco, Cajon de Letellier
	AO89	MT776496	Phyllotis vaccarum	Hap_011	Argentina	Pampa Palauco, Cajon de Letellier
	CL16	MT776473	Phyllotis posticalis_chilensis	Hap_033	Argentina	Jujuy: 4 km south from Abra Pampa on route 9
CMI6999	CL49	MT776469	Phyllotis posticalis_chilensis	Hap_034	Argentina	25 km west from Susques
CMI7132	CL63	MT776487	Phyllotis vaccarum	Hap_035	Argentina	Cortaderas, a 95 km O de Fiambalá, sobre R. 60
CMI7134	CL71	MT776488	Phyllotis vaccarum	Hap_035	Argentina	Cortaderas, a 95 km O de Fiambalá, sobre R. 60
CMI7177	CL77	MT776486	Phyllotis vaccarum	Hap_035	Argentina	Cortaderas, a 95 km O de Fiambalá, sobre R. 60
CMI7201	CL84	MT776489	Phyllotis vaccarum	Hap_036	Argentina	Cortaderas, a 95 km O de Fiambalá, sobre R. 60
UACH9124	GD1358	PQ295377	Phyllotis vaccarum	Hap_042	Chile	Camino Las Melosas, Sector Quebrada el Loro, San Jose de Maipo, Region Metropolitana
UACH9125	GD1359	PQ295378	Phyllotis vaccarum	Hap_043	Chile	Camino Las Melosas, Sector Quebrada el Loro, San Jose de Maipo, Region Metropolitana
UACH9126	GD1374	PQ295379	Phyllotis vaccarum	Hap_044	Chile	Camino Las Melosas, Sector Quebrada el Loro, San Jose de Maipo, Region Metropolitana
UACH9127	GD1376	PQ295380	Phyllotis vaccarum	Hap_042	Chile	Lo Valdes, San Jose de Maipo, Region Metropolitana
UACH9128	GD1626	PQ295381	Phyllotis vaccarum	Hap_045	Chile	San Fernando, Region Ohiggins
UACH9085	GD1749	PQ295382	Phyllotis darwini	Hap_046	Chile	Constitucion, Region Maule
UACH9129	GD2026	PQ295383	Phyllotis vaccarum	Hap_036	Chile	San Pedro de Atacama, Ruta B-241 km 177, Region Antofagasta
UACH9130	GD2029	PQ295384	Phyllotis vaccarum	Hap_047	Chile	San Pedro de Atacama, Ruta B-241 km 177, Region Antofagasta
UACH9104	GD2045	PQ295385	Phyllotis posticalis_chilensis	Hap_048	Chile	San Pedro de Atacama, Ruta 27 km 33, Region Antofagasta
UACH9131	GD2050	PQ295386	Phyllotis vaccarum	Hap_049	Chile	San Pedro de Atacama, Ruta 27 km 33, Region Antofagasta
UACH9132	GD2059	PQ295387	Phyllotis vaccarum	Hap_050	Chile	San Pedro de Atacama, Ruta 27 km 45.5, Region Antofagasta
UACH9133	GD2060	PQ295388	Phyllotis vaccarum	Hap_051	Chile	San Pedro de Atacama, Ruta 27 km 45.5, Region Antofagasta
UACH9134	GD2061	PQ295389	Phyllotis vaccarum	Hap_050	Chile	San Pedro de Atacama, Ruta 27 km 45.5, Region Antofagasta
UACH8559	GD2068	OR799595	Phyllotis vaccarum	Hap_035	Chile	San Pedro de Atacama, Ruta 27 km 33, Region Antofagasta
UACH9135	GD2069	PQ295390	Phyllotis vaccarum	Hap_035	Chile	San Pedro de Atacama, Ruta 27 km 33, Region Antofagasta
UACH9136	GD2070	PQ295391	Phyllotis vaccarum	Hap_035	Chile	San Pedro de Atacama, Ruta 27 km 33, Region Antofagasta
UACH8582	GD2082	MT183678	Phyllotis vaccarum	Hap_036	Chile	3 km SO Refugio Aguadas de Zorritas, PN Llullaillaco, Region Antofagasta
UACH8583	GD2083	MT183679	Phyllotis vaccarum	Hap_052	Chile	3 km SO Refugio Aguadas de Zorritas, PN Llullaillaco, Region Antofagasta
UACH8584	GD2084	MT183680	Phyllotis vaccarum	Hap_053	Chile	3 km SO Refugio Aguadas de Zorritas, PN Llullaillaco, Region Antofagasta
UACH8516	GD2093	MT183682	Phyllotis vaccarum	Hap_054	Chile	PN Llullaillaco, Campamento base este, Region Antofagasta
UACH8517	GD2094	MT183676	Phyllotis vaccarum	Hap_055	Chile	PN Llullaillaco, Campamento base este, Region Antofagasta
UACH8518	GD2095	MT183681	Phyllotis vaccarum	Hap_036	Chile	PN Llullaillaco, Campamento base este, Region Antofagasta
UACH8519	GD2096	OR810742	Phyllotis vaccarum	Hap_056	Chile	PN Llullaillaco, Campamento base este, Region Antofagasta
UACH8291	GD2097	MT183683	Phyllotis vaccarum	Hap_057	Chile	Cumbre Volcan Llullaillaco, Region Antofagasta
UACH8520	GD2099	OR810743	Phyllotis vaccarum	Hap_058	Chile	PN Llullaillaco, Campamento base este, Region Antofagasta
UACH8521	GD2100	OR799606	Phyllotis vaccarum	Hap_059	Chile	PN Llullaillaco, Campamento base este, Region Antofagasta
UACH9137	GD2103	PQ295392	Phyllotis vaccarum	Hap_060	Chile	PN Llullaillaco, Campamento base norte, Region Antofagasta
UACH9138	GD2104	PQ295393	Phyllotis vaccarum	Hap_061	Chile	PN Llullaillaco, Campamento base norte, Region Antofagasta
UACH9139	GD2105	PQ295394	Phyllotis vaccarum	Hap_062	Chile	PN Llullaillaco, Campamento base norte, Region Antofagasta
UACH9087	GD2117	PQ295395	Phyllotis limatus	Hap_063	Chile	Quillahuasa, Quebrada Tarapaca, Region Tarapaca
UACH8560	GD2118	OR799603	Phyllotis magister	Hap_064	Chile	Quillahuasa, Quebrada Tarapaca, Region Tarapaca
UACH8585	GD2120	OR799599	Phyllotis limatus	Hap_065	Chile	Quillahuasa, Quebrada Tarapaca, Region Tarapaca
UACH9088	GD2121	PQ295396	Phyllotis limatus	Hap_066	Chile	Quillahuasa, Quebrada Tarapaca, Region Tarapaca
UACH8586	GD2133	OR799609	Phyllotis limatus	Hap_067	Chile	Huarasina, Quebrada Tarapaca, Region Tarapaca
UACH8561	GD2134	OR799608	Phyllotis limatus	Hap_068	Chile	Huarasina, Quebrada Tarapaca, Region Tarapaca
UACH8562	GD2135	OR799610	Phyllotis limatus	Hap_067	Chile	Huarasina, Quebrada Tarapaca, Region Tarapaca
UACH8563	GD2150	OR799612	Phyllotis limatus	Hap_069	Chile	Quebrada de Camarones, Camarones, Ruta A-345, Region Arica y Parinacota
UACH8564	GD2155	OR799613	Phyllotis limatus	Hap_069	Chile	Quebrada de Camarones, Camarones, Ruta A-345, Region Arica y Parinacota

UACH8587	GD2174	OR799611	Phyllotis limatus	Hap_070	Chile	Quebrada de Camarones, Camarones, Ruta A-345, Region Arica y Parinacota
UACH8588	GD2175	OR799601	Phyllotis vaccarum	Hap_071	Chile	RN La Chimba, Antofagasta, Region Antofagasta
UACH9086	GD2219	PQ295397	Phyllotis darwini	Hap_072	Chile	Quebrada Cachina, Ruta B 980, Taltal,
UACH9089	GD2226	PQ295398	Phyllotis limatus	Hap_073	Chile	Chusmiza, Qda de Ocharaza, Region Arica y Parinacota
UACH9090	GD2227	PQ295399	Phyllotis limatus	Hap_074	Chile	Chusmiza, Qda de Ocharaza, Region Arica y Parinacota
UACH9091	GD2228	PQ295400	Phyllotis limatus	Hap_075	Chile	Chusmiza, Qda de Ocharaza, Region Arica y Parinacota
UACH9092	GD2229	PQ295401	Phyllotis limatus	Hap_076	Chile	Chusmiza, Qda de Ocharaza, Region Arica y Parinacota
UACH9093	GD2230	PQ295402	Phyllotis posticalis_chilensis	Hap_077	Chile	Chusmiza, Qda de Ocharaza, Region Arica y Parinacota
UACH9094	GD2231	PQ295403	Phyllotis limatus	Hap_063	Chile	Chusmiza, Qda de Ocharaza, Region Arica y Parinacota
UACH9095	GD2232	PQ295404	Phyllotis limatus	Hap_063	Chile	Chusmiza, Qda de Ocharaza, Region Arica y Parinacota
UACH9096	GD2233	PQ295405	Phyllotis limatus	Hap_078	Chile	Chusmiza, Qda de Ocharaza, Region Arica y Parinacota
UACH9097	GD2234	PQ295406	Phyllotis limatus	Hap_079	Chile	Chusmiza, Qda de Ocharaza, Region Arica y Parinacota
UACH8565	GD2241	OR799607	Phyllotis limatus	Hap_080	Chile	Chusmiza, Qda de Ocharaza, Region Arica y Parinacota
UACH9098	GD2242	PQ295407	Phyllotis limatus	Hap_080	Chile	Chusmiza, Qda de Ocharaza, Region Arica y Parinacota
UACH9100	GD2243	PQ295408	Phyllotis magister	Hap_081	Chile	Chusmiza, Qda de Ocharaza, Region Arica y Parinacota
UACH9101	GD2244	PQ295409	Phyllotis magister	Hap_064	Chile	Chusmiza, Qda de Ocharaza, Region Arica y Parinacota
UACH8566	GD2245	OR799598	Phyllotis limatus	Hap_063	Chile	Chusmiza, Qda de Ocharaza, Region Arica y Parinacota
UACH9105	GD2251	PQ295410	Phyllotis posticalis_chilensis	Hap_082	Chile	Turuma, Laguna Cotaculco, Colchane, Region Tarapaca
UACH9106	GD2252	PQ295411	Phyllotis posticalis_chilensis	Hap_083	Chile	Turuma, Laguna Cotaculco, Colchane, Region Tarapaca
UACH9107	GD2254	PQ295412	Phyllotis posticalis_chilensis	Hap_084	Chile	Turuma, Laguna Cotaculco, Colchane, Region Tarapaca
UACH9108	GD2255	PQ295413	Phyllotis posticalis_chilensis	Hap_083	Chile	Turuma, Laguna Cotaculco, Colchane, Region Tarapaca
UACH9109	GD2256	PQ295414	Phyllotis posticalis_chilensis	Hap_085	Chile	Turuma, Laguna Cotaculco, Colchane, Region Tarapaca
UACH9110	GD2257	PQ295415	Phyllotis posticalis_chilensis	Hap_086	Chile	Turuma, Laguna Cotaculco, Colchane, Region Tarapaca
UACH9111	GD2258	PQ295416	Phyllotis posticalis_chilensis	Hap_087	Chile	Turuma, Laguna Cotaculco, Colchane, Region Tarapaca
UACH9112	GD2259	PQ295417	Phyllotis posticalis_chilensis	Hap_088	Chile	Turuma, Laguna Cotaculco, Colchane, Region Tarapaca
UACH9113	GD2260	PQ295418	Phyllotis posticalis_chilensis	Hap_089	Chile	Turuma, Laguna Cotaculco, Colchane, Region Tarapaca
UACH9114	GD2261	PQ295419	Phyllotis posticalis_chilensis	Hap_090	Chile	Turuma, Laguna Cotaculco, Colchane, Region Tarapaca
UACH9115	GD2285	PQ295420	Phyllotis posticalis_chilensis	Hap_091	Chile	Laguna Casiri, Region Arica y Parinacota
UACH9140	GD2287	PQ295421	Phyllotis vaccarum	Hap_092	Chile	Cobija, Ruta 1 km 119.5, Region Antofagasta
UACH9141	GD2288	PQ295422	Phyllotis vaccarum	Hap_093	Chile	Gatico, Quebrada Gatico, Region Antofagasta
UACH9142	GD2289	PQ295423	Phyllotis vaccarum	Hap_094	Chile	Cobija, Ruta 1 km 119.5, Region Antofagasta
UACH9143	GD2290	PQ295424	Phyllotis vaccarum	Hap_095	Chile	Cobija, Ruta 1 km 119.5, Region Antofagasta
UACH9144	GD2291	PQ295425	Phyllotis vaccarum	Hap_096	Chile	Cobija, Ruta 1 km 119.5, Region Antofagasta
UACH9102	GD2347	PQ295426	Phyllotis magister	Hap_097	Chile	Maria Elena, Puente Teresa, Region Antofagasta
UACH9103	GD2348	PQ295427	Phyllotis magister	Hap_098	Chile	Maria Elena, Puente Teresa, Region Antofagasta
UACH8567	GD2349	OR799602	Phyllotis magister	Hap_099	Chile	Maria Elena, Puente Teresa, Region Antofagasta
UACH9099	GD2350	PQ295428	Phyllotis limatus	Hap_100	Chile	Maria Elena, Puente Teresa, Region Antofagasta
UACH8995	GD2458	PQ295429	Phyllotis vaccarum	Hap_282	Chile	Elqui, Vicuña, Ruta 41 km 149
UACH9002	GD2465	PQ295430	Phyllotis vaccarum	Hap_283	Chile	Elqui, Vicuña, Ruta 41 km 149
UACH9004	GD2467	PQ295431	Phyllotis vaccarum	Hap_155	Chile	Elqui, Vicuña, Ruta 41 km 149
UACH9005	GD2468	PQ295432	Phyllotis vaccarum	Hap_283	Chile	Elqui, Vicuña, Ruta 41 km 149
UACH9006	GD2469	PQ295433	Phyllotis vaccarum	Hap_285	Chile	Elqui, Vicuña, Ruta 41 km 144.400
UACH9007	GD2470	PQ295434	Phyllotis vaccarum	Hap_284	Chile	Elqui, Vicuña, Ruta 41 km 144.400
MACN-Ma29426	JPJ1213	MT776490	Phyllotis vaccarum	Hap_112	Argentina	Salta, Cuesta del Obispo
MACN-Ma29428	JPJ1286	MT776492	Phyllotis vaccarum	Hap_113	Argentina	Agua del Gauchi
MACN-Ma29421	JPJ1331	MT776472	Phyllotis posticalis_chilensis	Hap_114	Argentina	Salta, km ENE de Rodeo Pampa, km 59 de Ruta Provincial Nº 7
MACN-Ma29427	JPJ1421	MT776491	Phyllotis vaccarum	Hap_115	Argentina	Laguna Blanca
MACN-Ma29429	JPJ2155	MT776477	Phyllotis camiari	Hap_116	Argentina	Córdoba, Pampa de Achala
MACN-Ma29430	JPJ2156	MT776476	Phyllotis camiari	Hap_117	Argentina	Córdoba, Pampa de Achala
MACN-Ma29610	JPJ2226	PQ295435	Phyllotis vaccarum	Hap_118	Argentina	Famantina, Cueva de Diaz
MACN-Ma29419	JPJ2449	MT776470	Phyllotis posticalis_chilensis	Hap_119	Argentina	9 km al NW de Lizoite
MACN-Ma29420	JPJ2450	MT776471	Phyllotis posticalis_chilensis	Hap_119	Argentina	9 km al NW de Lizoite
MACN-Ma29587	JPJ2507	PQ295436	Phyllotis posticalis_chilensis	Hap_120	Argentina	Cochinoca, Guairazul
MACN-Ma29588	JPJ2510	PQ295437	Phyllotis posticalis_chilensis	Hap_121	Argentina	Jujuy, Dpto. Cochinoca. Guairazul
MACN-Ma29597	JPJ2532	PQ295438	Phyllotis posticalis_chilensis	Hap_122	Argentina	Jujuy, Dpto. Cochinoca. Guairazul
MACN-Ma29598	JPJ2535	PQ295439	Phyllotis posticalis_chilensis	Hap_121	Argentina	Jujuy, Dpto. Cochinoca. Guairazul
MACN-Ma27436	JPJ2536	PQ295440	Phyllotis posticalis_chilensis	Hap_121	Argentina	Jujuy, Dpto. Cochinoca. Guairazul
MACN-Ma29422	JPJ2704	MT776474	Phyllotis posticalis_chilensis	Hap_123	Argentina	Jujuy, Rachaite, ruta provincial 74
MACN-Ma29423	JPJ2705	MT776475	Phyllotis posticalis_chilensis	Hap_123	Argentina	Jujuy, Rachaite, ruta provincial 74

MACN-Ma31211	JPJ2766	PQ295441	Phyllotis vaccarum	Hap_124	Argentina	El Penon, La Vichina, La Rioja
MACN-Ma31207	JPJ2773	PQ295442	Phyllotis vaccarum	Hap_125	Argentina	El Penon, La Vichina, La Rioja
MACN-Ma31212	JPJ2780	PQ295443	Phyllotis vaccarum	Hap_124	Argentina	El Penon, La Vichina, La Rioja
MACN-Ma31208	JPJ2839	PQ295444	Phyllotis vaccarum	Hap_126	Argentina	La Vichina, La Rioja, Laguna Brava
MACN-Ma31213	JPJ2845	PQ295445	Phyllotis vaccarum	Hap_127	Argentina	La Vichina, La Rioja, Santo Domingo
MACN-Ma31214	JPJ2847	PQ295446	Phyllotis vaccarum	Hap_128	Argentina	La Vichina, La Rioja, Santo Domingo
MACN-Ma31209	JPJ2877	PQ295447	Phyllotis vaccarum	Hap_129	Argentina	La Vichina, La Rioja, Laguna Brava
MACN-Ma31210	JPJ2878	PQ295448	Phyllotis vaccarum	Hap_130	Argentina	La Vichina, La Rioja, Laguna Brava
MACN-Ma31215	JPJ2886	PQ295449	Phyllotis vaccarum	Hap_012	Argentina	La Vichina, La Rioja, Santo Domingo
MACN-Ma29424	JPJ624	KF442273	Phyllotis caprinus	Hap_134	Argentina	Barcena, Jujuy
MACN-Ma29425	JPJ640	MT776508	Phyllotis caprinus	Hap_131	Argentina	Barcena, Jujuy
	LCM10794	AY956704	Phyllotis andium	Hap_135	Peru	Lima, Oyon, Oyon
	LCM1156	AY956735	Phyllotis vaccarum	Hap_042	Chile	Region Metropolitana, El Yeso
	LCM1157	AY956736	Phyllotis vaccarum	Hap_042	Chile	Region Metropolitana, El Yeso
	LCM1161	AY956737	Phyllotis xanthopygus	Hap_136	Chile	Aysen, Chile Chico
	LCM1715	MZ460911	Phyllotis vaccarum	Hap_095	Chile	Mejillones, Antofagasta
	LCM1737	AY956739	Phyllotis vaccarum	Hap_036	Chile	Antofagasta, Tocopilla, Desembocadura Rio Loa
	LCM1780	AF484211	Phyllotis vaccarum	Hap_057	Chile	Antofagasta, Toconao
	LCM1794	MZ460910	Phyllotis vaccarum	Hap_036	Chile	Talabre
	LCM1804	AY956716	Phyllotis magister	Hap_137	Chile	El Loa, Ojo Opache, Calama
	LCM1813	AY956717	Phyllotis magister	Hap_137	Chile	El Loa, Ojo Opache, Calama
	LCM1829	AF484210	Phyllotis vaccarum	Hap_138	Chile	Talabre
	LCM1894	AY956718	Phyllotis magister	Hap_137	Chile	El Loa, Ojo Opache, Calama
	LCM2488	AY956722	Phyllotis darwini	Hap_139	Chile	Coquimbo, Petorca, Los Molles
	LCM2509	AY956726	Phyllotis darwini	Hap_140	Chile	Coquimbo, Petorca, Los Molles
	LSUMZ27823	AY956719	Phyllotis magister	Hap_141	Peru	Arequipa, 35 km E Arequipa
	LSUMZ27830	AF484214	Phyllotis magister	Hap_142	Peru	Arequipa, 35 km E Arequipa
	LSUMZ27832	AY956721	Phyllotis magister	Hap_141	Peru	Arequipa, 35 km E Arequipa
	LSUMZ27841	AF484208	Phyllotis limatus	Hap_063	Peru	Arequipa, 38 km E arequipa
LSUMZ27820	M1399	MT183677	Phyllotis limatus	Hap_213	Peru	13 km E Arequipa
	MACN26378	KT024789	Phyllotis nogalaris	Hap_145	Argentina	Quebrada Alumbriojo, aprox. 8 km al NE de Santa Ana
	MFS1324	U86833	Phyllotis xanthopygus	Hap_147	Argentina	Rio Negro, Comallo
UACH8554	MQC343	PQ295555	Phyllotis vaccarum	Hap_148	Chile	Ruinas Incas, Laguna de Santa Rosa, PN Nevado Tres Cruces, Region Atacama
UACH9145	MQC344	PQ295450	Phyllotis vaccarum	Hap_148	Chile	Ruinas Incas, Laguna de Santa Rosa, PN Nevado Tres Cruces, Region Atacama
UACH9146	MQC345	PQ295451	Phyllotis vaccarum	Hap_148	Chile	Ruinas Incas, Laguna de Santa Rosa, PN Nevado Tres Cruces, Region Atacama
UACH9147	MQC346	PQ295452	Phyllotis vaccarum	Hap_149	Chile	Ruinas Incas, Laguna de Santa Rosa, PN Nevado Tres Cruces, Region Atacama
UACH9148	MQC347	PQ295453	Phyllotis vaccarum	Hap_150	Chile	Ruinas Incas, Laguna de Santa Rosa, PN Nevado Tres Cruces, Region Atacama
UACH9149	MQC350	PQ295454	Phyllotis vaccarum	Hap_148	Chile	Ruinas Incas, Laguna de Santa Rosa, PN Nevado Tres Cruces, Region Atacama
UACH9150	MQC352	PQ295455	Phyllotis vaccarum	Hap_151	Chile	Refugio Maricunga, Laguna de Santa Rosa, PN Nevado Tres Cruces, Region Atacama
UACH9151	MQC353	PQ295456	Phyllotis vaccarum	Hap_149	Chile	Ruinas Incas, Laguna de Santa Rosa, PN Nevado Tres Cruces, Region Atacama
UACH9152	MQC354	PQ295457	Phyllotis vaccarum	Hap_152	Chile	Ruinas Incas, Laguna de Santa Rosa, PN Nevado Tres Cruces, Region Atacama
UACH9153	MQC355	PQ295458	Phyllotis vaccarum	Hap_153	Chile	Refugio Maricunga, Laguna de Santa Rosa, PN Nevado Tres Cruces, Region Atacama
UACH9154	MQC357	PQ295459	Phyllotis vaccarum	Hap_154	Chile	Refugio Laguna Verde, Region Atacama
UACH9155	MQC358	PQ295460	Phyllotis vaccarum	Hap_151	Chile	Refugio Laguna Verde, Region Atacama
UACH8568	MQC359	OR799573	Phyllotis vaccarum	Hap_155	Chile	Refugio Atacama, Volcan Ojos del Salado, Region Atacama
UACH8569	MQC360	OR799569	Phyllotis vaccarum	Hap_125	Chile	Refugio Atacama, Volcan Ojos del Salado, Region Atacama
UACH8570	MQC361	OR799574	Phyllotis vaccarum	Hap_155	Chile	Refugio Atacama, Volcan Ojos del Salado, Region Atacama
UACH8571	MQC362	OR799614	Phyllotis vaccarum	Hap_156	Chile	Refugio Atacama, Volcan Ojos del Salado, Region Atacama
UACH8572	MQC364	OR799593	Phyllotis vaccarum	Hap_155	Chile	Refugio Atacama, Volcan Ojos del Salado, Region Atacama
UACH8573	MQC365	OR799594	Phyllotis vaccarum	Hap_155	Chile	Refugio Atacama, Volcan Ojos del Salado, Region Atacama
UACH9156	MQC368	PQ295461	Phyllotis vaccarum	Hap_035	Chile	Gachi, Sector Guatin, Margen Rio Puritama, Region Antofagasta
UACH9157	MQC369	PQ295462	Phyllotis vaccarum	Hap_047	Chile	Gachi, Sector Guatin, Margen Rio Puritama, Region Antofagasta
UACH8574	MQC371	OR799566P	Phyllotis posticalis_chilensis	Hap_157	Chile	Volcan Aucanquilcha, Region Antofagasta
UACH9116	MQC372	PQ295463	Phyllotis posticalis_chilensis	Hap_158	Chile	Volcan Aucanquilcha, Region Antofagasta
UACH9117	MQC373	PQ295464	Phyllotis posticalis_chilensis	Hap_159	Chile	Volcan Aucanquilcha, Region Antofagasta
UACH9118	MQC374	PQ295465	Phyllotis posticalis_chilensis	Hap_160	Chile	Volcan Aucanquilcha, Region Antofagasta
UACH9119	MQC375	PQ295466	Phyllotis posticalis_chilensis	Hap_161	Chile	Volcan Aucanquilcha, Region Antofagasta
UACH9120	MQC376	PQ295467	Phyllotis posticalis_chilensis	Hap_162	Chile	Volcan Aucanquilcha, Region Antofagasta
UACH9121	MQC377	PQ295468	Phyllotis posticalis_chilensis	Hap_159	Chile	Volcan Aucanquilcha, Region Antofagasta

UACH9122	MQC378	PQ295469	Phyllotis posticalis_chilensis	Hap_158	Chile	Volcan Aucanquilcha, Region Antofagasta
UACH9123	MQC379	PQ295470	Phyllotis posticalis_chilensis	Hap_159	Chile	Volcan Aucanquilcha, Region Antofagasta
UACH9158	MQC382	PQ295471	Phyllotis vaccarum	Hap_163	Chile	Campamento Volcan Acamarachi, Region Antofagasta
UACH8556	MQC383	OR799591	Phyllotis vaccarum	Hap_164	Chile	Campamento Volcan Acamarachi, Region Antofagasta
UACH9159	MQC386	PQ295472	Phyllotis vaccarum	Hap_164	Chile	Campamento Volcan Acamarachi, Region Antofagasta
UACH9160	MQC391	PQ295473	Phyllotis vaccarum	Hap_035	Chile	Campamento Salar de Pular, Region Antofagasta
UACH8525	MQC392	OR799578	Phyllotis vaccarum	Hap_035	Chile	Campamento Salar de Pular, Region Antofagasta
UACH9161	MQC393	PQ295474	Phyllotis vaccarum	Hap_035	Chile	Campamento Salar de Pular, Region Antofagasta
UACH8526	MQC394	OR799588	Phyllotis vaccarum	Hap_059	Chile	Campamento Salar de Pular, Region Antofagasta
UACH9162	MQC395	PQ295475	Phyllotis vaccarum	Hap_035	Chile	Campamento Salar de Pular, Region Antofagasta
UACH8527	MQC396	OR799586	Phyllotis vaccarum	Hap_165	Chile	Campamento Salar de Pular, Region Antofagasta
UACH8528	MQC400	OR799572	Phyllotis vaccarum	Hap_166	Chile	Campamento Salar de Pular, Region Antofagasta
UACH8529	MQC401	OR799585	Phyllotis vaccarum	Hap_167	Chile	Campamento Salar de Pular, Region Antofagasta
UACH8530	MQC402	OR799587	Phyllotis vaccarum	Hap_165	Chile	Campamento Salar de Pular, Region Antofagasta
UACH8523	MQC403	OR799575	Phyllotis vaccarum	Hap_035	Chile	Campamento Alto Volcan Salin, Region Antofagasta
UACH8538	MQC404	OR810738	Phyllotis vaccarum	Hap_168	Chile	Cumbre Volcan Salin, Region Antofagasta
UACH8539	MQC405	OR810739	Phyllotis vaccarum	Hap_169	Chile	Cumbre Volcan Salin, Region Antofagasta
UACH8540	MQC406	OR810740	Phyllotis vaccarum	Hap_059	Chile	Cumbre Volcan Salin, Region Antofagasta
UACH8541	MQC407	OR810741	Phyllotis vaccarum	Hap_167	Chile	Cumbre Volcan Salin, Region Antofagasta
UACH8542	MQC408	OR799580	Phyllotis vaccarum	Hap_049	Chile	Cumbre Volcan Salin, Region Antofagasta
UACH8543	MQC409	OR810736	Phyllotis vaccarum	Hap_169	Chile	Cumbre Volcan Salin, Region Antofagasta
UACH8544	MQC410	OR810737	Phyllotis vaccarum	Hap_170	Chile	Cumbre Volcan Salin, Region Antofagasta
UACH8545	MQC411	OR799581	Phyllotis vaccarum	Hap_049	Chile	Cumbre Volcan Salin, Region Antofagasta
UACH8532	MQC415	OR799579	Phyllotis vaccarum	Hap_035	Chile	Campamento Salar de Pular, Region Antofagasta
UACH8533	MQC418	OR799590	Phyllotis vaccarum	Hap_171	Chile	Campamento Salar de Pular, Region Antofagasta
UACH8534	MQC419	OR799576	Phyllotis vaccarum	Hap_035	Chile	Campamento Salar de Pular, Region Antofagasta
UACH8535	MQC420	OR799589	Phyllotis vaccarum	Hap_172	Chile	Campamento Salar de Pular, Region Antofagasta
UACH9163	MQC421	PQ295476	Phyllotis vaccarum	Hap_035	Chile	Campamento Salar de Pular, Region Antofagasta
UACH8536	MQC422	OR799583	Phyllotis vaccarum	Hap_173	Chile	Campamento Salar de Pular, Region Antofagasta
UACH9164	MQC423	PQ295477	Phyllotis vaccarum	Hap_035	Chile	Campamento Salar de Pular, Region Antofagasta
UACH8575	MQC425	OR799565	Phyllotis posticalis_chilensis	Hap_174	Chile	Pirca camino al Volcan Sairecabur, Region Antofagasta
UACH9083	MQC426	PQ295478	Phyllotis posticalis_chilensis	Hap_157	Chile	Campamento Volcan Colorado, Region Antofagasta
UACH9084	MQC427	PQ295479	Phyllotis posticalis_chilensis	Hap_157	Chile	Campamento Volcan Colorado, Region Antofagasta
UACH8537	MQC428	OR810731	Phyllotis vaccarum	Hap_059	Chile	Cumbre Volcan Pular
UACH9165	MQC429	PQ295480	Phyllotis vaccarum	Hap_057	Chile	Salar de Aguas Calientes, Region Antofagasta
UACH9166	MQC430	PQ295481	Phyllotis vaccarum	Hap_057	Chile	Salar de Aguas Calientes, Region Antofagasta
UACH9167	MQC431	PQ295482	Phyllotis vaccarum	Hap_057	Chile	Salar de Aguas Calientes, Region Antofagasta
UACH9168	MQC432	PQ295483	Phyllotis vaccarum	Hap_057	Chile	Salar de Aguas Calientes, Region Antofagasta
UACH8549	MQC433	OR799577	Phyllotis vaccarum	Hap_035	Chile	Salar de Aguas Calientes, Region Antofagasta
UACH9169	MQC434	PQ295484	Phyllotis vaccarum	Hap_057	Chile	Salar de Aguas Calientes, Region Antofagasta
UACH9170	MQC437	PQ295485	Phyllotis vaccarum	Hap_036	Chile	Salar de Aguas Calientes, Region Antofagasta
UACH8550	MQC438	OR799584	Phyllotis vaccarum	Hap_057	Chile	Salar de Aguas Calientes, Region Antofagasta
UACH8551	MQC439	OR799570	Phyllotis vaccarum	Hap_151	Chile	Salar de Aguas Calientes, Region Antofagasta
UACH8552	MQC440	OR799592	Phyllotis vaccarum	Hap_175	Chile	Campamento Base Volcan Copiapo, Region Atacama
UACH8553	MQC441	OR799604	Phyllotis vaccarum	Hap_007	Chile	Campamento Base Volcan Copiapo, Region Atacama
UACH8579	MQC442	OR799596	Phyllotis vaccarum	Hap_176	Chile	Refugio Laguna Verde, Region Atacama
UACH8557	MQC443	OR799597	Phyllotis vaccarum	Hap_176	Chile	Refugio Laguna Verde, Region Atacama
UACH9171	MQC451	PQ295486	Phyllotis vaccarum	Hap_177	Chile	Vallecitos (Colas de Zorro), Region Atacama
UACH8580	MQC452	OR799600	Phyllotis vaccarum	Hap_151	Chile	Vallecitos (Colas de Zorro), Region Atacama
UACH8576	MQC453	OR799571	Phyllotis vaccarum	Hap_151	Chile	Vallecitos (Colas de Zorro), Region Atacama
UACH9172	MQC454	PQ295487	Phyllotis vaccarum	Hap_151	Chile	Vallecitos (Colas de Zorro), Region Atacama
UACH9173	MQC455	PQ295488	Phyllotis vaccarum	Hap_151	Chile	Vallecitos (Colas de Zorro), Region Atacama
UACH9174	MQC465	PQ295489	Phyllotis vaccarum	Hap_177	Chile	Vallecitos (Colas de Zorro), Region Atacama
UACH8577	MQC466	OR799582	Phyllotis vaccarum	Hap_178	Chile	Vallecitos (Colas de Zorro), Region Atacama
UACH8581	MQC467	OR799605	Phyllotis vaccarum	Hap_179	Chile	Vallecitos (Colas de Zorro), Region Atacama
UACH9175	MQC468	PQ295490	Phyllotis vaccarum	Hap_177	Chile	Vallecitos (Colas de Zorro), Region Atacama
UACH8578	MQC469	OR799567	Phyllotis vaccarum	Hap_180	Chile	Vallecitos (Colas de Zorro), Region Atacama
UACH8558	MQC470	OR799568	Phyllotis vaccarum	Hap_180	Chile	Vallecitos (Colas de Zorro), Region Atacama
UACH9176	MQC471	PQ295491	Phyllotis vaccarum	Hap_151	Chile	Vallecitos (Colas de Zorro), Region Atacama

UACH8555	MQC476	OR810732	Phyllotis vaccarum	Hap_180	Chile	Cumbre Volcan Copiapo
UACH8546	MQC520	OR810733	Phyllotis vaccarum	Hap_035	Chile	Campamento Salar de Pular, Region Antofagasta
UACH8547	MQC521	OR810734	Phyllotis vaccarum	Hap_138	Chile	Campamento Salar de Pular, Region Antofagasta
UACH8548	MQC522	OR810735	Phyllotis vaccarum	Hap_054	Chile	Campamento Salar de Pular, Region Antofagasta
UACH9177	MQC581	PQ295492	Phyllotis vaccarum	Hap_181	Chile	Camino Las Melosas, Sector Quebrada el Loro, San Jose de Maipo, Region Metropolitana
UACH9178	MQC582	PQ295493	Phyllotis vaccarum	Hap_182	Chile	Camino Las Melosas, Sector Quebrada el Loro, San Jose de Maipo, Region Metropolitana
UACH9179	MQC583	PQ295494	Phyllotis vaccarum	Hap_183	Chile	Camino Las Melosas, Sector Quebrada el Loro, San Jose de Maipo, Region Metropolitana
UACH9180	MQC584	PQ295495	Phyllotis vaccarum	Hap_184	Chile	Camino Las Melosas, Sector Quebrada el Loro, San Jose de Maipo, Region Metropolitana
UACH9181	MQC585	PQ295496	Phyllotis vaccarum	Hap_182	Chile	Camino Las Melosas, Sector Quebrada el Loro, San Jose de Maipo, Region Metropolitana
UACH9182	MQC586	PQ295497	Phyllotis vaccarum	Hap_185	Chile	Camino Las Melosas, Sector Quebrada el Loro, San Jose de Maipo, Region Metropolitana
UACH9183	MQC587	PQ295498	Phyllotis vaccarum	Hap_186	Chile	Camino Las Melosas, Sector Quebrada el Loro, San Jose de Maipo, Region Metropolitana
UACH9184	MQC589	PQ295499	Phyllotis vaccarum	Hap_187	Chile	Camino Las Melosas, Sector Quebrada el Loro, San Jose de Maipo, Region Metropolitana
UACH9185	MQC590	PQ295500	Phyllotis vaccarum	Hap_188	Chile	Camino Las Melosas, Sector Quebrada el Loro, San Jose de Maipo, Region Metropolitana
UACH9186	MQC591	PQ295501	Phyllotis vaccarum	Hap_189	Chile	Camino Las Melosas, Sector Quebrada el Loro, San Jose de Maipo, Region Metropolitana
UACH9187	MQC593	PQ295502	Phyllotis vaccarum	Hap_190	Chile	Camino Las Melosas, Sector Quebrada el Loro, San Jose de Maipo, Region Metropolitana
UACH9188	MQC594	PQ295503	Phyllotis vaccarum	Hap_191	Chile	Camino Las Melosas, Sector Quebrada el Loro, San Jose de Maipo, Region Metropolitana
UACH9189	MQC595	PQ295504	Phyllotis vaccarum	Hap_042	Chile	Camino Las Melosas, Sector Quebrada el Loro, San Jose de Maipo, Region Metropolitana
UACH9190	MQC596	PQ295505	Phyllotis vaccarum	Hap_192	Chile	Camino Las Melosas, Sector Quebrada el Loro, San Jose de Maipo, Region Metropolitana
UACH9191	MQC597	PQ295506	Phyllotis vaccarum	Hap_193	Chile	Camino Las Melosas, Sector Quebrada el Loro, San Jose de Maipo, Region Metropolitana
UACH9192	MQC598	PQ295507	Phyllotis vaccarum	Hap_194	Chile	Camino Las Melosas, Sector Quebrada el Loro, San Jose de Maipo, Region Metropolitana
UACH9193	MQC599	PQ295508	Phyllotis vaccarum	Hap_195	Chile	Camino Las Melosas, Sector Quebrada el Loro, San Jose de Maipo, Region Metropolitana
	PNG1034	HM167913	Phyllotis xanthopygus	Hap_234	Argentina	Provincia Chubut, Las Plumas
	PNG1287	HM167914	Phyllotis xanthopygus	Hap_235	Argentina	Leleque, Cuadro La Portada
	PNG167	HM167905	Phyllotis xanthopygus	Hap_101	Argentina	Meseta Somuncurá, Campamento PNG
	PNG173	HM167906	Phyllotis xanthopygus	Hap_102	Argentina	Provincia Río Negro, Meseta Somuncurá, Subida del Naciente
	PNG197	HM167907	Phyllotis xanthopygus	Hap_103	Argentina	Provincia Chubut, Ea Talagapa
	PNG325	HM167908	Phyllotis xanthopygus	Hap_104	Argentina	Provincia Chubut, Ea Quichaura
	PNG342	HM167909	Phyllotis xanthopygus	Hap_105	Argentina	Ea Bajada del Guanaco
	PNG613	HM167910	Phyllotis xanthopygus	Hap_106	Argentina	Provincia Santa Cruz, Ea La Ensenada
CBF11168	PZ738	OR784649	Phyllotis posticalis_chilensis	Hap_019	Bolivia	Depto. Oruro, Campamento base volcán Acotango
CBF11172	PZ742	OR784658	Phyllotis posticalis_chilensis	Hap_019	Bolivia	Depto. Oruro, Geiser Sajama, Linea 1
CBF11173	PZ743	OR784643	Phyllotis posticalis_chilensis	Hap_020	Bolivia	Depto. Oruro, Sajama, mirador Monte Cielo
CBF11174	PZ744	OR784644	Phyllotis posticalis_chilensis	Hap_020	Bolivia	Depto. Oruro, Sajama, mirador Monte Cielo
CBF11175	PZ745	OR784647	Phyllotis posticalis_chilensis	Hap_021	Bolivia	Depto. Oruro, Sajama, mirador Monte Cielo
CBF11176	PZ746	OR784661	Phyllotis posticalis_chilensis	Hap_022	Bolivia	Depto. Oruro, Sajama, mirador Monte Cielo
CBF11177	PZ747	OR784645	Phyllotis posticalis_chilensis	Hap_020	Bolivia	Depto. Oruro, Sajama, mirador Monte Cielo
CBF11178	PZ748	OR784653	Phyllotis posticalis_chilensis	Hap_023	Bolivia	Depto. Oruro, Sajama, mirador Monte Cielo
CBF11187	PZ757	OR784646	Phyllotis posticalis_chilensis	Hap_020	Bolivia	Depto. Oruro, Sajama, mirador Monte Cielo
CBF11189	PZ759	OR784651	Phyllotis posticalis_chilensis	Hap_024	Bolivia	Depto. Oruro, Geiser Sajama, Linea 1
CBF11190	PZ760	OR784654	Phyllotis posticalis_chilensis	Hap_025	Bolivia	Depto. Oruro, Geiser Sajama, Linea 4
CBF11191	PZ761	OR784655	Phyllotis posticalis_chilensis	Hap_026	Bolivia	Depto. Oruro, Campamento base "alto" Volcán Parinacota
CBF11193	PZ763	OR784657	Phyllotis posticalis_chilensis	Hap_027	Bolivia	Depto. Oruro, Campamento base "alto" Volcán Parinacota
CBF11194	PZ764	OR784656	Phyllotis posticalis_chilensis	Hap_028	Bolivia	Depto. Oruro, Campamento base "alto" Volcán Parinacota
CBF11200	PZ770	OR784648	Phyllotis posticalis_chilensis	Hap_029	Bolivia	Depto. Oruro, Geiser Sajama, Linea 1
CBF11202	PZ772	OR784650	Phyllotis posticalis_chilensis	Hap_030	Bolivia	Depto. Oruro, Geiser Sajama, Linea 1
CBF11203	PZ773	OR784659	Phyllotis posticalis_chilensis	Hap_031	Bolivia	Depto. Oruro, Geiser Sajama, Linea 1
CBF11204	PZ774	OR784660	Phyllotis posticalis_chilensis	Hap_031	Bolivia	Depto. Oruro, Geiser Sajama, Linea 2
CBF11214	PZ784	OR784652	Phyllotis posticalis_chilensis	Hap_032	Bolivia	Depto. Oruro, Campamento base Volcán Sajama
	RAO03	MT776498	Phyllotis vaccarum	Hap_236	Argentina	Quebrada del Toro
	RAO1168	MT776468	Phyllotis posticalis_chilensis	Hap_237	Argentina	pre-puna: RAO 1168
	RAO125	MT776484	Phyllotis pehuenche	Hap_005	Argentina	Mendoza: 10 km al S de Las Leñas, margen del Río Salado
	RAO126	MT776482	Phyllotis pehuenche	Hap_016	Argentina	Mendoza: 10 km al S de Las Leñas, margen del Río Salado
	RAO130	MT776481	Phyllotis pehuenche	Hap_016	Argentina	Mendoza: 10 km al S de Las Leñas, margen del Río Salado
	RAO138	MT776483	Phyllotis pehuenche	Hap_238	Argentina	Mendoza: 10 km al S de Las Leñas, margen del Río Salado
	RAO140	MT776497	Phyllotis vaccarum	Hap_239	Argentina	Quebrada del Toro
	RAO141	MT776502	Phyllotis vaccarum	Hap_007	Argentina	Quebrada del Toro
	RAO144	MT776504	Phyllotis vaccarum	Hap_240	Argentina	Quebrada del Toro
	RAO145	MT776499	Phyllotis vaccarum	Hap_241	Argentina	Quebrada del Toro
	RCO1537	UACH8249	Phyllotis vaccarum	Hap_242	Chile	Los Queñes- Río Teno

RCO1538	UACH8250	Phyllotis vaccarum	Hap_243	Chile	Los Queñes- Río Teno
RCO1540	UACH8252	Phyllotis vaccarum	Hap_244	Chile	Los Queñes- Río Teno
RCO1567	UACH8255	Phyllotis vaccarum	Hap_245	Chile	La Buitrera, cerca de Codelco
RCO1568	UACH8256	Phyllotis vaccarum	Hap_246	Chile	La Buitrera, cerca de Codelco
RCO1569	UACH8257	Phyllotis vaccarum	Hap_247	Chile	La Buitrera, cerca de Codelco
RCO1628	UACH8280	Phyllotis vaccarum	Hap_248	Chile	Los Queñes- Río Teno
RCO1630	UACH8281	Phyllotis vaccarum	Hap_249	Chile	Los Queñes- Río Teno
RCO1631	UACH8282	Phyllotis vaccarum	Hap_250	Chile	Los Queñes- Río Teno
UFJPAC20	AF484209	Phyllotis camiari	Hap_258	Argentina	Córdoba, Pampa de Achala
UP111	AY275128	Phyllotis camiari	Hap_259	Argentina	Córdoba, Pampa de Achala
UP349	MZ298892	Phyllotis pehuenche	Hap_260	Argentina	Neuquen, Sierra Cuchillo Cura
UP389	MZ298886	Phyllotis pehuenche	Hap_261	Argentina	Neuquen, Piedra del aguila
UP390	MZ298887	Phyllotis pehuenche	Hap_262	Argentina	Neuquen, Piedra del aguila
UP391	MZ298888	Phyllotis pehuenche	Hap_263	Argentina	Neuquen, Piedra del aguila
UP393	MZ298889	Phyllotis pehuenche	Hap_264	Argentina	Neuquen, Piedra del aguila
UP398	MZ298860	Phyllotis pehuenche	Hap_264	Argentina	Neuquen, Cerrito Pinon
UP399	MZ298861	Phyllotis pehuenche	Hap_266	Argentina	Neuquen, Cerrito Pinon
UP400	MZ298862	Phyllotis pehuenche	Hap_262	Argentina	Neuquen, Cerrito Pinon
UP401	MZ298863	Phyllotis pehuenche	Hap_267	Argentina	Neuquen, Cerrito Pinon
UP402	MZ298864	Phyllotis pehuenche	Hap_262	Argentina	Neuquen, Cerrito Pinon
UP403	MZ298865	Phyllotis pehuenche	Hap_257	Argentina	Neuquen, Cerrito Pinon
UP405	MZ298866	Phyllotis pehuenche	Hap_265	Argentina	Neuquen, Cerrito Pinon
UP406	MZ298867	Phyllotis pehuenche	Hap_265	Argentina	Neuquen, Cerrito Pinon
UP407	MZ298868	Phyllotis pehuenche	Hap_264	Argentina	Neuquen, Cerrito Pinon
UP408	MZ298869	Phyllotis pehuenche	Hap_265	Argentina	Neuquen, Cerrito Pinon
UP409	MZ298870	Phyllotis pehuenche	Hap_268	Argentina	Neuquen, Cerrito Pinon
UP410	MZ298871	Phyllotis pehuenche	Hap_268	Argentina	Neuquen, Cerrito Pinon
UP411	MZ298872	Phyllotis pehuenche	Hap_268	Argentina	Neuquen, Cerrito Pinon
UP412	MZ298873	Phyllotis pehuenche	Hap_269	Argentina	Neuquen, Cerrito Pinon
UP413	MZ298874	Phyllotis pehuenche	Hap_270	Argentina	Neuquen, Cerrito Pinon
UP414	MZ298875	Phyllotis pehuenche	Hap_265	Argentina	Neuquen, Cerrito Pinon
UP416	MZ298876	Phyllotis pehuenche	Hap_271	Argentina	Neuquen, Cerrito Pinon
UP417	MZ298877	Phyllotis pehuenche	Hap_265	Argentina	Neuquen, Cerrito Pinon
UP418	MZ298878	Phyllotis pehuenche	Hap_272	Argentina	Neuquen, Cerrito Pinon
UP419	MZ298879	Phyllotis pehuenche	Hap_265	Argentina	Neuquen, Cerrito Pinon
UP420	MZ298880	Phyllotis pehuenche	Hap_265	Argentina	Neuquen, Cerrito Pinon
UP421	MZ298881	Phyllotis pehuenche	Hap_265	Argentina	Neuquen, Cerrito Pinon
UP425	MZ298883	Phyllotis pehuenche	Hap_273	Argentina	Neuquen, Las Coloradas
UP426	MZ298884	Phyllotis pehuenche	Hap_265	Argentina	Neuquen, Las Coloradas
UP427	MZ298885	Phyllotis pehuenche	Hap_265	Argentina	Neuquen, Las Coloradas
UP433	MZ298882	Phyllotis pehuenche	Hap_264	Argentina	Neuquen, Cerrito Pinon
UP434	MZ298859	Phyllotis pehuenche	Hap_274	Argentina	Neuquen, Canadon Santo Domingo
UP437	MZ298890	Phyllotis pehuenche	Hap_274	Argentina	Sierra Cuchillo Cura, Las Lajas, Ea. La Portena
UP438	MZ298891	Phyllotis pehuenche	Hap_274	Argentina	Sierra Cuchillo Cura, Las Lajas, Ea. La Portena
UP440	MZ298893	Phyllotis pehuenche	Hap_274	Argentina	Sierra Cuchillo Cura, Las Lajas, Ea. La Portena
UP441	MZ298894	Phyllotis pehuenche	Hap_275	Argentina	Sierra Cuchillo Cura, Las Lajas, Ea. La Portena
UP468	MT183684	Phyllotis vaccarum	Hap_276	Argentina	Neuquén,Buta Ranquil
UP474	MZ460908	Phyllotis vaccarum	Hap_222	Argentina	Mendoza, Los Frisos, El Zampal
UP478	MZ298848	Phyllotis pehuenche	Hap_257	Argentina	Mendoza, Bardas Blancas
UP480	MZ298849	Phyllotis pehuenche	Hap_261	Argentina	Mendoza, Bardas Blancas
UP483	MZ298850	Phyllotis pehuenche	Hap_277	Argentina	Mendoza, Bardas Blancas
UP484	MZ298851	Phyllotis pehuenche	Hap_278	Argentina	Mendoza, Bardas Blancas
UP485	MZ298852	Phyllotis pehuenche	Hap_257	Argentina	Mendoza, Bardas Blancas
UP486	MZ298853	Phyllotis pehuenche	Hap_277	Argentina	Mendoza, Bardas Blancas
UP487	MZ298854	Phyllotis pehuenche	Hap_257	Argentina	Mendoza, Bardas Blancas
UP489	MZ298855	Phyllotis pehuenche	Hap_017	Argentina	Mendoza, Bardas Blancas
UP490	MZ298856	Phyllotis pehuenche	Hap_257	Argentina	Mendoza, Bardas Blancas
UP497	MZ298857	Phyllotis pehuenche	Hap_279	Argentina	Mendoza, Laguna de la Nina Encantada
UP505	MZ298858	Phyllotis pehuenche	Hap_016	Argentina	Mendoza, Laguna de la Nina Encantada

	UP611	MZ460909	Phyllotis vaccarum	Hap_215	Argentina	Catamarca, Laguna Blanca
	UP613	MZ460912	Phyllotis vaccarum	Hap_112	Argentina	Salta, Alto Cachi
	UP614	MT183688	Phyllotis vaccarum	Hap_217	Argentina	Salta, Alto Cachi
	UP645	MT183689	Phyllotis posticalis_chilensis	Hap_237	Argentina	Camino a Garganta del Diablo
	UWBM72232	AY956734	Phyllotis vaccarum	Hap_280	Argentina	49.2 km N central Mendoza
	VPT2318	AY956714	Phyllotis pearsoni	Hap_281	Peru	Distribucion P pearsoni
AC21		AY956733	Phyllotis camiari	Hap_001	Argentina	Córdoba, Pampa de Achala
MVZ182703		AF108693	Phyllotis xanthopygus	Hap_002	Argentina	10 km de Comallo, Pilcaniyeu
		KY754115	Phyllotis posticalis_chilensis	Hap_003	Peru	Cusco, Pisac
AK13012		AY041190	Phyllotis vaccarum	Hap_007	Argentina	3 km W Refugio Militar Grl. Alvarado, Mendoza
AK13013		AY041191	Phyllotis vaccarum	Hap_013	Argentina	3 km W Refugio Militar Grl. Alvarado, Mendoza
NK95979		AY341046	Phyllotis limatus	Hap_014	Chile	Socoroma
CNP736		AY627299	Phyllotis anitae	Hap_015	Argentina	10 km by road south of Hualinchay
NK96038		AY746968	Phyllotis posticalis_chilensis	Hap_021	Chile	Lago Chungara
NK96039		AY746969	Phyllotis posticalis_chilensis	Hap_021	Chile	Lago Chungara
UACH4923		PQ295553	Phyllotis posticalis_chilensis	Hap_021	Chile	Lago Chungara
UACH4924		PQ295554	Phyllotis posticalis_chilensis	Hap_021	Chile	Lago Chungara
NK96006		AY341053	Phyllotis posticalis_chilensis	Hap_021	Chile	Putre
NK96040		AY746970	Phyllotis posticalis_chilensis	Hap_024	Chile	Lago Chungara
CML7542		GQ119625	Phyllotis anitae	Hap_037	Argentina	Parque Nacional Campo de los Alisos
FMNH107476		U86822	Phyllotis limatus	Hap_038	Peru	Moquegua, Torata
UACH4911		PQ295549	Phyllotis limatus	Hap_038	Chile	Parinacota
UACH4913		PQ295551	Phyllotis limatus	Hap_038	Chile	Parinacota
UACH4921		PQ295552	Phyllotis limatus	Hap_038	Chile	Putre
FMNH107606		U86830	Phyllotis posticalis_chilensis	Hap_039	Peru	Tarata, Tacna
FMNH107870		U86826	Phyllotis osilae	Hap_040	Peru	Puno, Chicuito, Ilave
FMNH133830		U86831	Phyllotis posticalis_chilensis	Hap_041	Chile	Chapiquina, 10 km S
UACH4912		PQ295550	Phyllotis limatus	Hap_063	Chile	Parinacota
SSUCMA00209		AY341043	Phyllotis limatus	Hap_063	Chile	Reserva Nacional Pampa del Tamarugal
NK95975		AY341047	Phyllotis limatus	Hap_063	Chile	Tambo de Zapahuira, Comuna Putre, Parinacota Province
UACH4910		PQ295548	Phyllotis limatus	Hap_065	Chile	Parinacota
		HM167911	Phyllotis xanthopygus	Hap_107	Argentina	Estancia Cerro Ventana
		HM167912	Phyllotis xanthopygus	Hap_108	Argentina	Estancia Cerro Ventana
		HM167915	Phyllotis xanthopygus	Hap_109	Argentina	Santa Cruz, Estancia Cerro del Paso
		HM167916	Phyllotis xanthopygus	Hap_110	Argentina	Estancia Los Manantiales
		HM167917	Phyllotis xanthopygus	Hap_111	Argentina	Chubut,Camarones
		JQ434420	Auliscomys boliviensis	Hap_132		OUTGROUP
		JQ434421	Auliscomys sublimis	Hap_133		OUTGROUP
UACH3933		PQ295544	Phyllotis xanthopygus	Hap_136	Argentina	Aysen, Chile Chico
UACH3934		PQ295545	Phyllotis xanthopygus	Hap_136	Argentina	Aysen, Chile Chico
UACH3935		PQ295546	Phyllotis xanthopygus	Hap_136	Argentina	Aysen, Chile Chico
LSUMZ27861		AF484212	Phyllotis posticalis_chilensis	Hap_143	Argentina	53 km E Arequipa
MACN26394		KT024804	Phyllotis tucumanus	Hap_146	Argentina	10 km al S de Hualinchay
PAM00006		AY341049	Phyllotis posticalis_chilensis	Hap_157	Chile	Arroyo Coya, Tocopilla
MSB133051		PQ295509	Phyllotis posticalis_chilensis	Hap_196	Bolivia	Cercanias Salitrera Mapocho
MSB236350		PQ295520	Phyllotis posticalis_chilensis	Hap_196	Bolivia	La Paz, Laguna Viscachani
MSB210456		PQ295510	Phyllotis posticalis_chilensis	Hap_197	Bolivia	Departamento de Oruro, Caracollo
MSB210457		PQ295511	Phyllotis posticalis_chilensis	Hap_197	Bolivia	Departamento de Oruro, Caracollo
MSB221141		PQ295512	Phyllotis posticalis_chilensis	Hap_198	Bolivia	Sotalaya; 25 km by rd NW Achacachi
MSB232422		PQ295513	Phyllotis posticalis_chilensis	Hap_199	Bolivia	Tarija, Serrania del Sama
MSB232427		PQ295515	Phyllotis posticalis_chilensis	Hap_199	Bolivia	Tarija, Serrania del Sama
MSB232428		PQ295516	Phyllotis posticalis_chilensis	Hap_199	Bolivia	Tarija, Serrania del Sama
MSB234923		PQ295517	Phyllotis posticalis_chilensis	Hap_200	Bolivia	Cochabamba, Tinkusiri, 17km E of Totora
MSB236347		PQ295519	Phyllotis posticalis_chilensis	Hap_200	Bolivia	La Paz, Laguna Viscachani
MSB68535		PQ295529	Phyllotis posticalis_chilensis	Hap_200	Bolivia	La Paz, MINA LOURDES
MSB236932		PQ295521	Phyllotis posticalis_chilensis	Hap_200	Bolivia	Tarija, 1 KM E OF ISCAYACHI, RIO TOMAYAPO
MSB232423		PQ295514	Phyllotis posticalis_chilensis	Hap_200	Bolivia	Tarija, Serrania del Sama
MSB236344		PQ295518	Phyllotis osilae	Hap_201	Bolivia	La Paz, Laguna Viscachani
MSB236946		PQ295522	Phyllotis posticalis_chilensis	Hap_202	Bolivia	Oruro, 7 KM S AND 4 KM E OF CRUCE VENTILLA

MSB67261	U86832	Phyllotis posticalis_chilensis	Hap_202	Bolivia	Tariija, 4.5 KM. E. OF ISCAYACHI 3750M. 21D 29 S. 64D 55' W
MSB53350	AY956738	Phyllotis posticalis_chilensis	Hap_203	Bolivia	Oruro, 1 KM W HUANCAROMA
MSB236974	PQ295523	Phyllotis posticalis_chilensis	Hap_203	Bolivia	Oruro, 1 KM W OF HUANCAROMA
MSB237236	PQ295524	Phyllotis caprinus	Hap_204	Bolivia	Chuquisaca, 2 km N of Tarabuco
MSB238568	PQ295525	Phyllotis caprinus	Hap_205	Bolivia	Cochabamba, 7.5 km SE Rodeo Curubamba
MSB240240	PQ295526	Phyllotis posticalis_chilensis	Hap_206	Bolivia	Potosi, 11 km SSE Betanzos, Estacion Exptl. Chinoli (IBTA)
MSB75256	PQ295534	Phyllotis posticalis_chilensis	Hap_206	Bolivia	Potosi, 11 km SSE Betanzos, Estacion Exptl. Chinoli (IBTA)
MSB240427	PQ295527	Phyllotis posticalis_chilensis	Hap_207	Bolivia	Oruro, ESTANCIA AGUA RICA, 40 KM E AND 22 KM S OF SAJAMA
MSB240457	PQ295528	Phyllotis posticalis_chilensis	Hap_208	Bolivia	La Paz, Challajipina
MSB69977	U86820	Phyllotis darwini	Hap_209	Chile	Limari, Parque Nacional Fray Jorge
MSB70558	PQ295530	Phyllotis posticalis_chilensis	Hap_210	Bolivia	Cochabamba, 7.5 km SE Rodeo Curubamba
MSB70563	PQ295531	Phyllotis posticalis_chilensis	Hap_210	Bolivia	La Paz, 8.5KM W OF SAN ANDREAS de MACHACA
MSB70564	PQ295532	Phyllotis posticalis_chilensis	Hap_211	Bolivia	La Paz, 8.5KM W OF SAN ANDREAS de MACHACA
MSB75225	PQ295533	Phyllotis posticalis_chilensis	Hap_212	Bolivia	Potosi, 11 km SSE Betanzos, Estacion Exptl. Chinoli (IBTA)
	MT183685	Phyllotis vaccarum	Hap_214	Argentina	Laguna Blanca
	MT183686	Phyllotis vaccarum	Hap_215	Argentina	Laguna Blanca
	MT183687	Phyllotis vaccarum	Hap_216	Argentina	Laguna Blanca
MUSM38651	KX793701	Phyllotis stenops	Hap_218	Bolivia	Luya, Cocabamba, Túen
MUSM38652	KX793702	Phyllotis stenops	Hap_219	Bolivia	Luya, Cocabamba, Túen
MUSM45095	KX793699	Phyllotis definitus	Hap_220	Peru	Ultu, Mina Pierina, Huaraz
MUSM45254	KX793700	Phyllotis definitus	Hap_220	Peru	Ultu, Mina Pierina, Huaraz
MUSN10787	AY956708	Phyllotis amicus	Hap_221	Peru	Chancay, Lachay
MSB210383	AY341052	Phyllotis posticalis_chilensis	Hap_223	Chile	Lago Chungara
NK96053	AY341050	Phyllotis posticalis_chilensis	Hap_224	Chile	Enquelga
NK96061	AY341051	Phyllotis posticalis_chilensis	Hap_225	Chile	Suricayo, Comuna Colchane
NK96199	AY746971	Phyllotis darwini	Hap_226	Chile	Fundo El Toyo, Comuna San José de Maipo
ORB125	AY956707	Phyllotis andium	Hap_227	Peru	Cajamarca, Cumbe Mayo
ORB14	AY956720	Phyllotis magister	Hap_228	Peru	Tarata, Tacna
ORB58	AY956712	Phyllotis gerbilus	Hap_229	Peru	Piura, Sechura
ORB59	AY956713	Phyllotis gerbilus	Hap_230	Peru	Piura, Sechura
ORB82	AY956730	Phyllotis posticalis_chilensis	Hap_231	Peru	Lima, Huarochiri, Casapalca
PAM00212	AY341045	Phyllotis limatus	Hap_232	Peru	Tignamar
PAM00315	AY341048	Phyllotis limatus	Hap_233	Peru	Alto Belén
SA02	AY956731	Phyllotis bonariensis	Hap_251	Argentina	Buenos Aires: Parque Provincial Ernesto Tronquist, Abra de La ventana
SA03	AY956732	Phyllotis bonariensis	Hap_252	Argentina	Buenos Aires: Parque Provincial Ernesto Tronquist, Abra de La ventana
SSUCMA00210	AY341044	Phyllotis limatus	Hap_253	Chile	Reserva Nacional Pampa del Tamarugal
	U03545	Auliscomys pictus	Hap_254		OUTGROUP
FMNH107615	U86821	Phyllotis limatus	Hap_255	Peru	Tarata, Tacna
UACH3936	PQ295547	Phyllotis xanthopygus	Hap_256	Argentina	Aysen, Chile Chico
	HM167822	Callomys musculinus	Hap_286		OUTGROUP
	GU553838	Auliscomys micropus	Hap_287		OUTGROUP

TABLE S2. Specimens of the genus *Phyllotis* included in the analyses of whole-genome sequence (WGS) data. Information for each specimen includes voucher number, taxonomic assignation, Country, and locality description.

Voucher number	Species	Country	Locality
JPJ2156	Phyllotis camiari	Argentina	Arg, Cord, Pampa de Achala
JPJ640	Phyllotis caprinus	Argentina	Arg, Jujuy, Barcena
GD1748	Phyllotis darwini	Chile	Ch, Maule, Curepto, Ruta J52K Km 4.6
GD1806	Phyllotis darwini	Chile	Ch, Coq, Tongoy, Ruta D-440 km 4.5
GD1863	Phyllotis darwini	Chile	Ch, O'H, Pichilemu, Cahuil
GD1876	Phyllotis darwini	Chile	Ch, O'H, Pichilemu, Cahuil
GD2176	Phyllotis darwini	Chile	Taltal, Paposo, Ruta 1 km 95.700
GD2177	Phyllotis darwini	Chile	Taltal, Paposo, Ruta 1 km 95.700
GD2178	Phyllotis darwini	Chile	Taltal, Paposo, Ruta 1 km 95.700
GD2179	Phyllotis darwini	Chile	Taltal, Paposo, Ruta 1 km 95.700
GD2180	Phyllotis darwini	Chile	Taltal, Paposo, Ruta 1 km 95.700
GD2181	Phyllotis darwini	Chile	Taltal, Paposo, Ruta 1 km 95.700
GD2183	Phyllotis darwini	Chile	Taltal, Paposo, Ruta 1 km 95.700
GD2184	Phyllotis darwini	Chile	Taltal, Paposo, Ruta 1 km 95.700
GD2185	Phyllotis darwini	Chile	Taltal, Paposo, Ruta 1 km 95.700
GD2189	Phyllotis darwini	Chile	Taltal, Paposo, Ruta 1 km 95.700
GD2190	Phyllotis darwini	Chile	Taltal, Paposo, Ruta 1 km 95.700
GD2191	Phyllotis darwini	Chile	Taltal, Paposo, Ruta 1 km 95.700
GD2197	Phyllotis darwini	Chile	Taltal, Paposo, Ruta 1 km 85.200
GD2205	Phyllotis darwini	Chile	Taltal, Paposo, Ruta 1 km 85.200
GD2218	Phyllotis darwini	Chile	Taltal, Quebrada Cachina, Ruta B 980
GD2219	Phyllotis darwini	Chile	Taltal, Quebrada Cachina, Ruta B 980
GD2222	Phyllotis darwini	Chile	Taltal, Quebrada Cachina, Ruta B 980
GD2223	Phyllotis darwini	Chile	Taltal, Quebrada Cachina, Ruta B 980
GD2224	Phyllotis darwini	Chile	Taltal, Quebrada Cachina, Ruta B 980
GD2117	Phyllotis limatus	Chile	Huara, Quebrada de Tarapaca, Quillahuasa
GD2120	Phyllotis limatus	Chile	Huara, Quebrada de Tarapaca, Quillahuasa
GD2121	Phyllotis limatus	Chile	Huara, Quebrada de Tarapaca, Quillahuasa
GD2133	Phyllotis limatus	Chile	Huara, Quebrada de Tarapaca, Huarasiña
GD2134	Phyllotis limatus	Chile	Huara, Quebrada de Tarapaca, Huarasiña
GD2135	Phyllotis limatus	Chile	Huara, Quebrada de Tarapaca, Huarasiña
GD2150	Phyllotis limatus	Chile	Camarones, Quebrada de Camarones, Ruta A-345 Km 28
GD2155	Phyllotis limatus	Chile	Camarones, Quebrada de Camarones, Ruta A-345 Km 28

GD2174	Phyllotis limatus	Chile	Camarones, Quebrada de Camarones, Ruta A-345 Km 20.700
GD2226	Phyllotis limatus	Chile	Huara, Chusmiza
GD2228	Phyllotis limatus	Chile	Huara, Chusmiza
GD2229	Phyllotis limatus	Chile	Huara, Chusmiza
GD2231	Phyllotis limatus	Chile	Huara, Chusmiza
GD2232	Phyllotis limatus	Chile	Huara, Chusmiza
GD2233	Phyllotis limatus	Chile	Huara, Chusmiza
GD2234	Phyllotis limatus	Chile	Huara, Chusmiza
GD2241	Phyllotis limatus	Chile	Huara, entrada Chusmiza
GD2242	Phyllotis limatus	Chile	Huara, entrada Chusmiza
GD2245	Phyllotis limatus	Chile	Huara, entrada Chusmiza
GD2350	Phyllotis limatus	Chile	María Elena, Río Loa
GD2118	Phyllotis magister	Chile	Huara, Quebrada de Tarapaca, Quillahuasa
GD2119	Phyllotis magister	Chile	Huara, Quebrada de Tarapaca, Quillahuasa
GD2243	Phyllotis magister	Chile	Huara, entrada Chusmiza
GD2244	Phyllotis magister	Chile	Huara, entrada Chusmiza
GD2349	Phyllotis magister	Chile	María Elena, Río Loa
GD2351	Phyllotis magister	Chile	María Elena, Río Loa
JPJ2187	Phyllotis nogalaris	Argentina	Jujuy, Quebrada Alumbriojo
JPJ2196	Phyllotis nogalaris	Argentina	Jujuy, Quebrada Alumbriojo
JPJ979	Phyllotis anitae	Argentina	Tucuman, Hualinchay
JPJ994	Phyllotis anitae	Argentina	Tucuman, Hualinchay
RAO124	Phyllotis pehuenche	Argentina	Mendoza, Valle Hermoso
RAO125	Phyllotis pehuenche	Argentina	Mendoza, Valle Hermoso
RAO126	Phyllotis pehuenche	Argentina	Mendoza, Valle Hermoso
GD2230	Phyllotis chilensis-posticalis	Chile	Huara, Chusmiza
GD2251	Phyllotis chilensis-posticalis	Chile	Colchane, Turuna, Cota Kulco
GD2252	Phyllotis chilensis-posticalis	Chile	Colchane, Turuna, Cota Kulco
GD2253	Phyllotis chilensis-posticalis	Chile	Colchane, Turuna, Cota Kulco
GD2254	Phyllotis chilensis-posticalis	Chile	Colchane, Turuna, Cota Kulco
GD2255	Phyllotis chilensis-posticalis	Chile	Colchane, Turuna, Cota Kulco
GD2256	Phyllotis chilensis-posticalis	Chile	Colchane, Turuna, Cota Kulco
GD2257	Phyllotis chilensis-posticalis	Chile	Colchane, Turuna, Cota Kulco
GD2258	Phyllotis chilensis-posticalis	Chile	Colchane, Turuna, Cota Kulco

GD2259	Phyllotis chilensis-posticalis	Chile	Colchane, Turuna, Cota Kulco
GD2260	Phyllotis chilensis-posticalis	Chile	Colchane, Turuna, Cota Kulco
GD2261	Phyllotis chilensis-posticalis	Chile	Colchane, Turuna, Cota Kulco
GD2285	Phyllotis chilensis-posticalis	Chile	Putre, Ruta A-119 km 15.900, Laguna Casiri Macho
MQC371	Phyllotis chilensis-posticalis	Chile	Linea 4, Volcan Aucalquilcha
MQC372	Phyllotis chilensis-posticalis	Chile	Linea 4, Volcan Aucalquilcha
MQC373	Phyllotis chilensis-posticalis	Chile	Linea 5, Volcan Aucalquilcha
MQC374	Phyllotis chilensis-posticalis	Chile	Linea 5, Volcan Aucalquilcha
MQC375	Phyllotis chilensis-posticalis	Chile	Linea 5, Volcan Aucalquilcha
MQC376	Phyllotis chilensis-posticalis	Chile	Linea 4, Volcan Aucalquilcha
MQC377	Phyllotis chilensis-posticalis	Chile	Linea 5, Volcan Aucalquilcha
MQC379	Phyllotis chilensis-posticalis	Chile	Linea 5, Volcan Aucalquilcha
MQC425	Phyllotis chilensis-posticalis	Chile	Pirca Camino al Sairecabur
MQC426	Phyllotis chilensis-posticalis	Chile	Campamento Volcan Colorado
MQC427	Phyllotis chilensis-posticalis	Chile	Campamento Volcan Colorado
GD2068	Phyllotis vaccarum	Chile	San Pedro de Atacama, Ruta 27 CH km 33
GD2093	Phyllotis vaccarum	Chile	Antofagasta, PN Llullaillaco, volcan Llullaillaco, campamento base Este
GD2094	Phyllotis vaccarum	Chile	Antofagasta, PN Llullaillaco, volcan Llullaillaco, campamento base Este
GD2095	Phyllotis vaccarum	Chile	Antofagasta, PN Llullaillaco, volcan Llullaillaco, campamento base Este
GD2096	Phyllotis vaccarum	Chile	Antofagasta, PN Llullaillaco, volcan Llullaillaco, campamento base Este
GD2097	Phyllotis vaccarum	Chile	PN Llullaillaco, volcan Llullaillaco, cumbre de Llullaillaco
GD2099	Phyllotis vaccarum	Chile	Antofagasta, PN Llullaillaco, volcan Llullaillaco, campamento base Este
GD2100	Phyllotis vaccarum	Chile	Antofagasta, PN Llullaillaco, volcan Llullaillaco, campamento base Este
GD2101	Phyllotis vaccarum	Chile	Antofagasta, PN Llullaillaco, volcan Llullaillaco, campamento base Este
MQC359	Phyllotis vaccarum	Chile	Campo base - Refugio Atacama
MQC360	Phyllotis vaccarum	Chile	Campo base - Refugio Atacama
MQC361	Phyllotis vaccarum	Chile	Campo base - Refugio Atacama
MQC362	Phyllotis vaccarum	Chile	Campo base - Refugio Atacama
MQC364	Phyllotis vaccarum	Chile	Campo base - Refugio Atacama
MQC365	Phyllotis vaccarum	Chile	Campo base - Refugio Atacama
MQC383	Phyllotis vaccarum	Chile	Campamento base Volcan Acamarachi
MQC390	Phyllotis vaccarum	Chile	Campamento Salar de Pular
MQC392	Phyllotis vaccarum	Chile	Campamento Salar de Pular
MQC394	Phyllotis vaccarum	Chile	Campamento Salar de Pular

MQC396	Phyllotis vaccarum	Chile	Campamento Salar de Pular
MQC400	Phyllotis vaccarum	Chile	Campamento Salar de Pular
MQC401	Phyllotis vaccarum	Chile	Campamento Salar de Pular
MQC402	Phyllotis vaccarum	Chile	Campamento Salar de Pular
MQC403	Phyllotis vaccarum	Chile	Campamento Alto Volcan Salin
MQC404	Phyllotis vaccarum	Chile	Cumbre Volcan Salin
MQC405	Phyllotis vaccarum	Chile	Cumbre Volcan Salin
MQC406	Phyllotis vaccarum	Chile	Cumbre Volcan Salin
MQC408	Phyllotis vaccarum	Chile	Cumbre Volcan Salin
MQC410	Phyllotis vaccarum	Chile	Cumbre Volcan Salin
MQC411	Phyllotis vaccarum	Chile	Cumbre Volcan Salin
MQC414	Phyllotis vaccarum	Chile	Campamento Salar de Pular
MQC415	Phyllotis vaccarum	Chile	Campamento Salar de Pular
MQC418	Phyllotis vaccarum	Chile	Campamento Salar de Pular
MQC419	Phyllotis vaccarum	Chile	Campamento Salar de Pular
MQC420	Phyllotis vaccarum	Chile	Campamento Salar de Pular
MQC422	Phyllotis vaccarum	Chile	Campamento Salar de Pular
MQC428	Phyllotis vaccarum	Chile	Cumbre Volcan Pular
MQC433	Phyllotis vaccarum	Chile	Salar de Aguas Calientes
MQC438	Phyllotis vaccarum	Chile	Salar de Aguas Calientes
MQC439	Phyllotis vaccarum	Chile	Salar de Aguas Calientes
MQC440	Phyllotis vaccarum	Chile	Campamento Base Volcan Copiapo
MQC441	Phyllotis vaccarum	Chile	Campamento Base Volcan Copiapo
MQC443	Phyllotis vaccarum	Chile	Refugio Laguna Verde
MQC453	Phyllotis vaccarum	Chile	Vallecitos, Pajas Grandes (Colas de Zorro)
MQC466	Phyllotis vaccarum	Chile	Vallecitos, Pajas Grandes (Colas de Zorro)
MQC469	Phyllotis vaccarum	Chile	Vallecitos, Pajas Grandes (Colas de Zorro)
MQC470	Phyllotis vaccarum	Chile	Vallecitos, Pajas Grandes (Colas de Zorro)
MQC476	Phyllotis vaccarum	Chile	Cumbre Volcan Copiapo
MQC520	Phyllotis vaccarum	Chile	Cumbre Volcan Pular
MQC521	Phyllotis vaccarum	Chile	Cumbre Volcan Pular
MQC522	Phyllotis vaccarum	Chile	Cumbre Volcan Pular
PPA385	Phyllotis xanthopygus	Argentina	Arg, Santa Cruz, Rio Chico, Cajon del Rio Oro
PPA421	Phyllotis xanthopygus	Argentina	Arg, Santa Cruz, Rio Chico, La peninsula

PPA551	Phyllotis xanthopygus	Argentina	Arg, Santa Cruz, Rio Chico Valle del Rio Oro
PPA555	Phyllotis xanthopygus	Argentina	Arg, Santa Cruz, Rio Chico Valle del Rio Oro

TABLE S3. Mean *p*-distance calculated based on *cytb* variation between pairs of lineages within nominal species of *Phyllotis* that were identified by the species delimitation analyses. Mean intra-lineage distance estimates are shown in bold in the diagonal. Standard errors (SE) of each pairwise distance estimate is shown above the diagonal. Internal clades are named according to their position (top-to-bottom) along branch-tips of the tree depicted in Figure 2. Specimens in the group labeled '*P. vaccarum**' carry *cytb* haplotypes that group with haplotypes of *P. limatus*, even though whole-genome sequence data confirmed their identity as *P. vaccarum* (Storz et al. 2024).

	1	2	3	4	5	6	7	8	9	10	11	12	13
1. *P. caprinus* 1	**0.379**	0.770	1.047	1.208	1.232	1.139	1.094	1.101	0.945	0.911	1.016	0.901	0.927
2. *P. caprinus* 2	5.591	**1.623**	1.133	1.265	1.064	1.046	1.116	1.049	1.014	1.037	1.122	0.831	0.886
3. *P. darwini* 1	11.976	11.549	**4.061**	0.679	1.173	1.178	1.114	1.152	1.115	1.062	1.145	0.979	1.048
4. *P. darwini* 2	13.285	12.360	5.399	**0.915**	1.128	1.100	1.201	1.224	1.239	1.230	1.133	1.110	1.127
5. *P. magister* 1	11.772	10.637	10.508	10.762	**0.166**	0.348	0.987	0.960	1.064	1.070	1.156	0.999	1.010
6. *P. magister* 2	11.277	10.620	10.924	10.895	1.809	**1.507**	0.940	0.869	1.023	1.041	1.053	0.996	1.004
7. *P. posticalis* 2	10.113	10.674	12.616	12.890	10.824	10.470	**--**	0.661	0.790	0.820	0.953	1.141	1.153
8. *P. posticalis* 4	9.987	9.925	11.863	12.079	8.951	8.483	3.995	**--**	0.680	0.637	0.986	1.049	1.010
9. *P. chilensis*	9.475	9.799	12.294	12.717	11.321	10.934	6.122	5.116	**0.888**	0.386	0.981	1.003	1.021
10. *P. posticalis* 3	9.438	9.785	12.397	12.633	11.511	11.126	6.367	5.056	2.069	**1.132**	0.993	1.022	1.057
11. *P. posticalis* 1	10.407	10.025	12.491	12.421	10.051	9.919	8.701	7.818	8.994	9.001	**--**	1.215	1.228
12. *P. vaccarum*	8.090	6.592	12.165	12.400	10.171	10.352	10.886	9.724	9.566	9.578	11.592	**1.982**	0.526
13. *P. vaccarum**	7.806	6.516	12.072	11.992	9.546	9.643	10.502	9.017	8.941	9.330	11.109	3.084	**0.544**

TABLE S4. Mean pairwise *p*-distances computed based on WGS data among the 11 nominal species of *Phyllotis* included in the genomic assessment (see Material and Methods and Figure 5).

	1	2	3	4	5	6	7	8	9	10	11
1. *P. camiari*		0.0100	0.0111	0.0114	0.0096	0.0115	0.0141	0.0101	0.0103	0.0095	0.0100
2. *P. caprinus*	0.0100		0.0116	0.0120	0.0091	0.0121	0.0146	0.0106	0.0107	0.0088	0.0104
3. *P. darwini north*	0.0111	0.0116		0.0048	0.0110	0.0078	0.0134	0.0115	0.0110	0.0110	0.0111
4. *P. darwini south*	0.0114	0.0120	0.0048		0.0114	0.0082	0.0139	0.0118	0.0114	0.0113	0.0115
5. *P. limatus*	0.0096	0.0091	0.0110	0.0114		0.0115	0.0139	0.0098	0.0095	0.0060	0.0099
6. *P. magister*	0.0115	0.0121	0.0078	0.0082	0.0115		0.0139	0.0119	0.0115	0.0114	0.0115
7. *P. tucumanus*	0.0141	0.0146	0.0134	0.0139	0.0139	0.0139		0.0146	0.0141	0.0136	0.0142
8. *P. pehuenche*	0.0101	0.0106	0.0115	0.0118	0.0098	0.0119	0.0146		0.0108	0.0097	0.0070
9. *P. chilensis-posticalis*	0.0103	0.0107	0.0110	0.0114	0.0095	0.0115	0.0141	0.0108		0.0099	0.0105
10. *P. vaccarum*	0.0095	0.0088	0.0110	0.0113	0.0060	0.0114	0.0136	0.0097	0.0099		0.0099
11. *P. xanthopygus*	0.0100	0.0104	0.0111	0.0115	0.0099	0.0115	0.0142	0.0070	0.0105	0.0099	

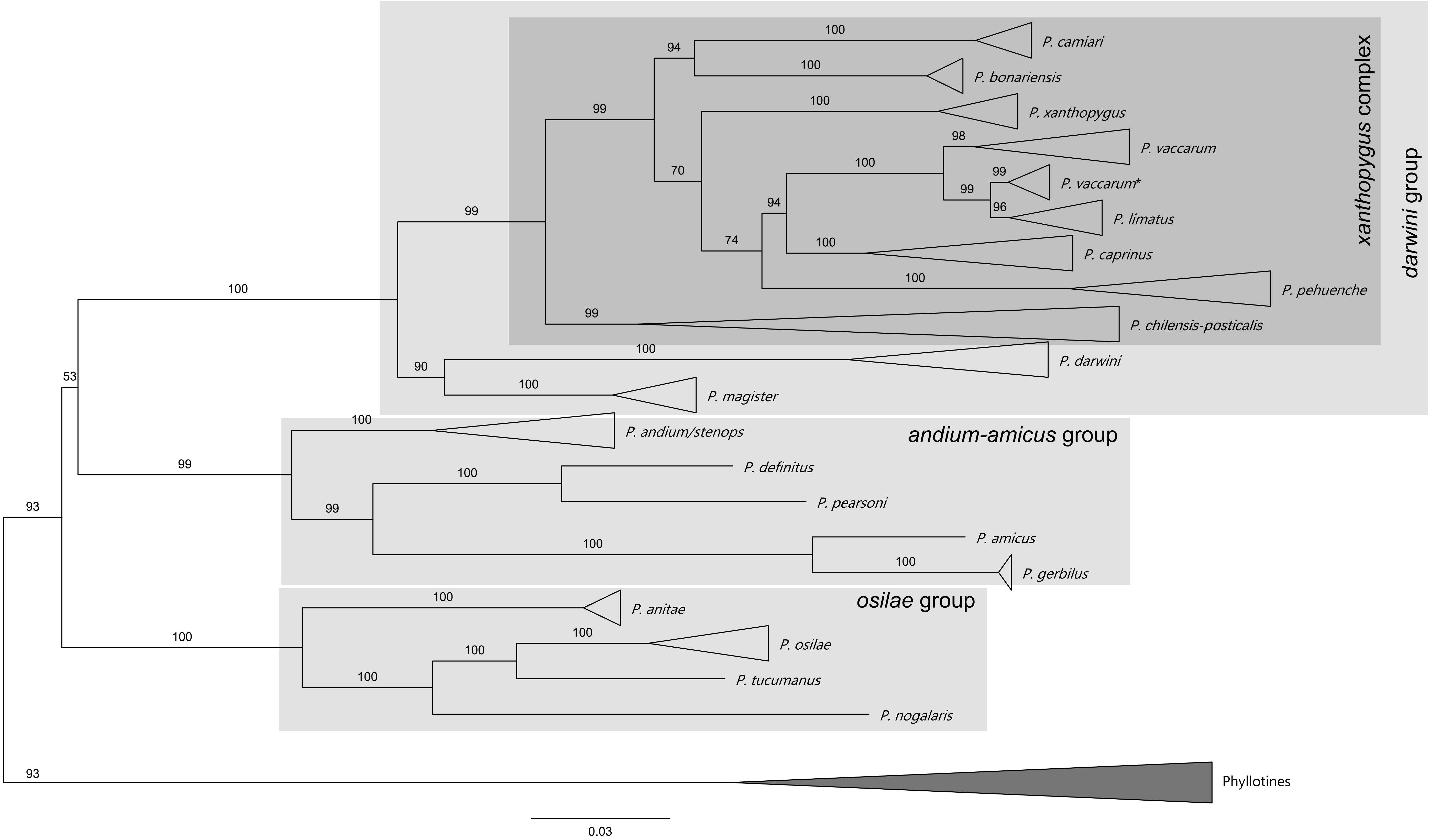

FIGURE S1. Phylogenetic relationships of *Phyllotis* species estimated from a ML analysis of 454 cytb gene sequences. ML consensus tree (ln = -7890.962). Supports for internal nodes are shown as ultrafast bootstrap values. Clades at species level are collapsed to simplify visualization of relationships. Specimens in the clade labeled *P. vaccarum** carry cytb haplotypes that are sister to the set of *P. limatus* haplotypes, even though whole-genome sequence data confirmed their identity as *P. vaccarum* (Storz et al., 2024).

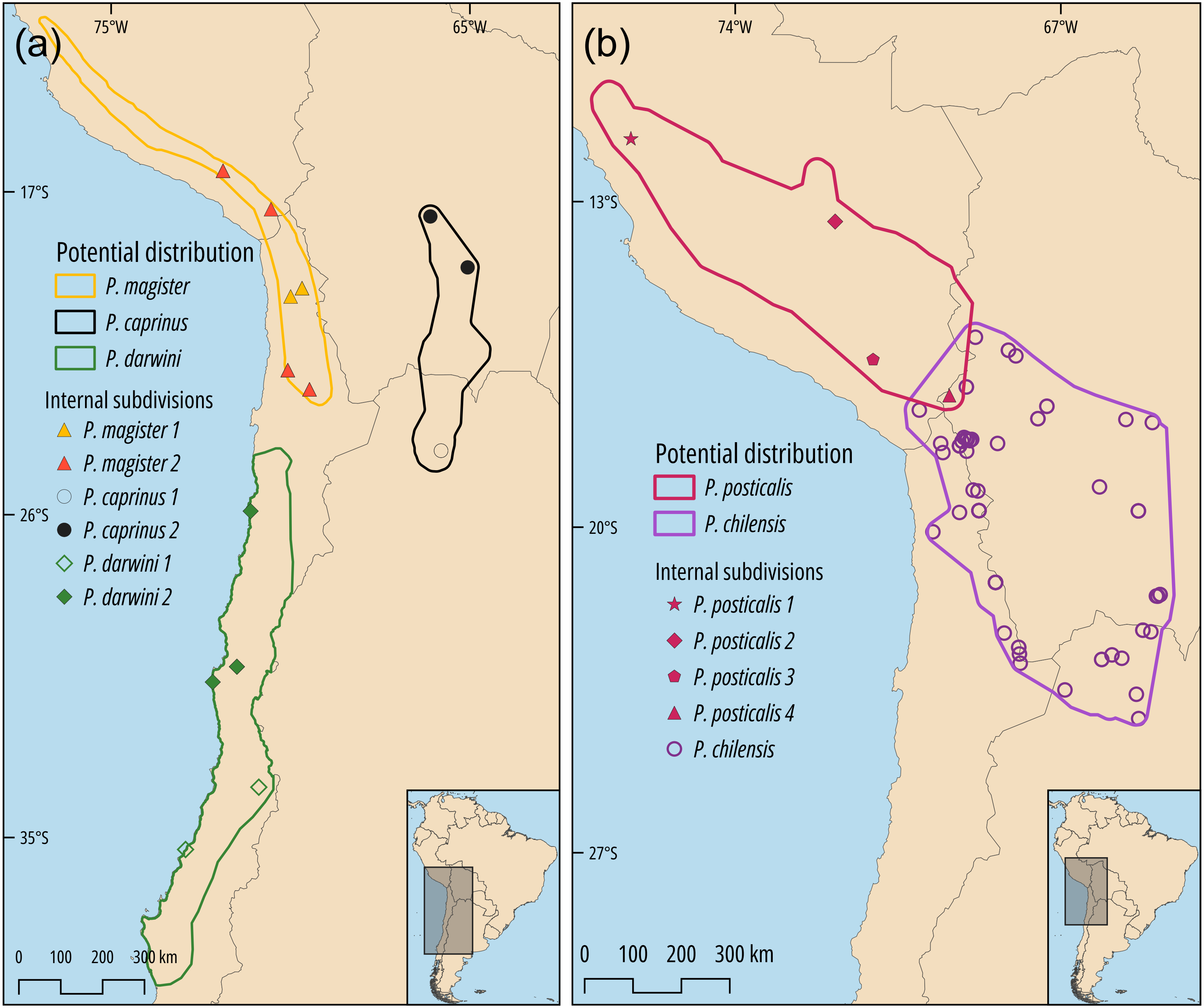

FIGURE S2. Geographic distributions of highly divergent, internal subdivisions within several species in the *Phyllotis darwini* group. A) Distribution of representatives of genetically distinct subdivisions within *Phyllotis caprinus*, *P. darwini*, and *P. magister*. B) Distribution of representatives of internal clades of "*P. chilensis-posticalis*". For reasons explained in the text, we regard representatives of the clade with the southernmost distribution as "*P. chilensis*", whereas representatives of the more northern, Peruvian clades are provisionally labelled "*P. posticalis* 1, 2, 3, and 4". Numbers for each clade correspond to their position in the tree shown in Figure 2.
